# Prioritising Content for Continuing Education in Non-Invasive Ventilation Care: A Needs Assessment Survey of Intensive Care Unit Nurses

**DOI:** 10.3390/healthcare14193326

**Published:** 2026-10-06

**Authors:** Seung Eun Jung, Yoon Ju Noh, Jae Yong Yoo

**Affiliations:** 1 Department of Nursing, Chosun University Hospital, Gwangju 61453, Republic of Korea; 2022097@csuh.co.kr; 2 Graduate School, Chosun University, Gwangju 61452, Republic of Korea; noh_yj@chosun.ac.kr; 3 Department of Nursing, Chosun Nursing College, Gwangju 61453, Republic of Korea; 4 Department of Nursing, College of Medicine, Chosun University, Gwangju 61452, Republic of Korea

**Keywords:** noninvasive ventilation, critical care nursing, needs assessment, education, nursing, continuing, evidence-based practice

## Abstract

Background: The success of non-invasive ventilation (NIV) depends on bedside nursing judgement, yet nurses report insufficient preparation, and educational content is usually decided before the competencies nurses most need are identified. Objectives: To prioritise NIV competencies, derived for this study from three clinical practice guidelines, by educational need, and, as secondary aims, describe intensive care unit nurses’ perceived knowledge, self-efficacy and implementation climate for evidence-based practice and their correlates. Methods: A cross-sectional survey questionnaire was completed by 142 nurses in four intensive care units of a tertiary teaching hospital in the Republic of Korea in June and July 2026 (response rate 97.3%). Each of 35 competencies was rated for importance and present competency; priorities were derived by combining the Borich needs assessment with the Locus for Focus model, and their stability was examined by bootstrap resampling. Results: Item means were 5.47 (SD 2.05) for perceived knowledge and 5.53 (SD 2.20) for self-efficacy on a 10-point scale and 1.81 (SD 0.78) for implementation climate on a 4-point scale. Perceived knowledge and self-efficacy were higher among nurses with experience of NIV care and previous NIV education, and these associations remained after adjustment. Length of intensive care experience was not independently associated with either variable, and neither was correlated with implementation climate. Importance exceeded present competency for all 35 items. Seven items were identified as priority candidates; ventilator waveform monitoring was the only item selected by both methods, but its classification was not stable across resamples. Conclusions: The self-perceived priorities concentrated on monitoring and recognising treatment failure, competencies that require interpretation of changing physiological data. Needs assessment before programme design can inform the selection of content, provided that its self-report basis and the instability of items near classification thresholds are recognised.

## 1. Introduction

Nurses continue to develop clinical capability after licensure through in-service and continuing education, an established means of maintaining the knowledge and skills required for safe and effective care [1,2]. The content of this education, however, is commonly determined before the competencies nurses most need have been identified, and programme evaluations examine educational outcomes rather than the adequacy of the content selected. The consequences are greatest for clinical interventions that are technically demanding and to which nurses are exposed at differing frequencies across units.

Non-invasive ventilation (NIV) delivers positive pressure through a mask or other interface without an artificial airway and is widely used in acute respiratory failure. Its benefit is conditional on appropriate patient selection and timing [3,4], and its success depends on bedside nursing care that includes fitting and maintenance of the interface, monitoring of the response to treatment, and early recognition of treatment failure.

Nurses’ preparation for NIV care appears inadequate. An integrative review of nurses’ experiences of NIV reported that few nurses considered themselves sufficiently trained to deliver this care [5], and most intensive care unit (ICU) nurses surveyed had insufficient knowledge of NIV [6]. Educational interventions of relatively short duration have improved these outcomes. A simulation programme incorporating a brief mask-wearing experience improved ventilator nursing knowledge and self-efficacy among general ward nurses [7]; that population differs from ICU nurses in case exposure and baseline preparation, but the study shows that both outcomes respond to education. Such studies, however, evaluate a programme once its content has been determined, and do not identify which content nurses need most beforehand. The issue is more pressing where preparation during undergraduate education is limited. A review of the curricula of the 185 four-year nursing programmes accredited in Korea as of 2016 found that a dedicated critical care nursing subject was offered in 42 (22.7%) [8]. Curricula may have changed since that review, but it indicated that preparation for NIV care rests largely on in-service and continuing education after licensure.

Individual knowledge and self-efficacy, however, are unlikely to be sufficient on their own. Whether a practice is enacted at the bedside depends on the capability of the individual and on the organisational conditions in which the practice is expected to occur. Implementation climate denotes employees’ shared perception of the extent to which use of a specific practice is expected, supported and rewarded within their unit [9], and the Implementation Climate Scale was developed to measure this perception for evidence-based practice in health services [10]. Unit implementation climate has a direct effect on nurses’ evidence-based practice behaviour [11], whereas individual characteristics account for only a small proportion of the variance in how nurses perceive that climate [12]. The distinction is relevant to needs assessment because a competency gap may arise from a deficit in individual capability, an unsupportive climate, or both, and education addresses only the first. Implementation climate was accordingly included as a secondary construct, measured as individual nurses’ perceptions of their unit, to examine whether self-appraised capability and the perceived organisational conditions for evidence-based practice varied together. The relationship between implementation climate and ICU nurses’ capability in a specific clinical practice such as NIV care has not, to our knowledge, been examined.

Establishing educational priorities further requires a method that separates competencies of high importance from those that are simply performed at a low level. The needs assessment model proposed by Borich [13] weights the discrepancy between perceived importance and current performance by the importance rating, and the Locus for Focus model [14] locates each competency in one of four quadrants defined by importance and discrepancy; competencies appearing in both solutions are designated the highest priority [15]. This combined approach has been applied to prioritise educational needs among clinical nurses [16] and school nurses [17]. Previous work on NIV nursing education has either described nurses’ knowledge, experiences and perceptions of NIV [5,6] or evaluated programmes whose content had already been fixed [7,18,19]. Few studies have assessed ICU nurses’ educational needs for NIV care, and, to our knowledge, none has derived the competencies from clinical practice guidelines, rated them for both importance and present performance, and prioritised them by educational need.

The primary aim of this study was to determine priorities for evidence-based NIV nursing education from the discrepancy between the perceived importance of each guideline-derived competency and the level at which nurses rated themselves as currently able to perform it. Secondary aims were to describe ICU nurses’ perceived knowledge of and self-efficacy in NIV care and their perceptions of the unit implementation climate for evidence-based practice, identify the participant characteristics associated with these variables, and examine the relationships among them. The analyses addressing the secondary aims were exploratory. The findings are intended to inform the selection of content for NIV nursing education programmes for ICU nurses.

## 2. Materials and Methods

### 2.1. Study Design

A cross-sectional, descriptive survey design was used. The study is reported in accordance with the Strengthening the Reporting of Observational Studies in Epidemiology (STROBE) checklist for cross-sectional studies (Supplementary File S2).

### 2.2. Study Participants and Setting

The study was conducted in the medical, surgical, neurological and emergency ICUs of an 849-bed tertiary teaching hospital in a metropolitan city in the Republic of Korea. The four units together comprise 73 beds and are staffed by registered nurses; there are no dedicated respiratory therapists, and ventilator management at the bedside is a nursing responsibility. NIV is initiated on medical prescription in all four units under a common institutional protocol so that indications, initial ventilator settings and weaning criteria do not differ across the units. Participants were recruited by convenience sampling. Nurses were eligible if they had worked in their current ICU for at least three months and provided direct patient care independently. Unit managers and other nurses not engaged in direct patient care were excluded. During the recruitment period, 151 nurses were employed in the four units and 146 met the eligibility criteria. The nursing administration office and the ICU nurse managers were informed of the purpose and procedures of the study in advance and assisted with recruitment, but they were not present when questionnaires were distributed or returned (Section 2.4).

Sample size was estimated for the exploratory comparisons of perceived knowledge, self-efficacy and implementation climate across participant characteristics, because no established procedure exists for determining the sample required by the Borich needs assessment or the Locus for Focus model, which are descriptive ranking procedures and not inferential tests. With G*Power 3.1.9.7 (Heinrich Heine University Düsseldorf, Düsseldorf, Germany), 126 participants were required for a one-way analysis of variance with four groups, an effect size of *f* = 0.30 (no prior estimate being available in NIV nursing), α of 0.05 and power of 0.80. The largest requirement across the planned analyses was 128 for two-group comparisons (*d* = 0.50, two-tailed). Allowing for approximately 15% non-response or incomplete questionnaires, about 150 participants were sought, and questionnaires were distributed to all 146 eligible nurses. The adequacy of the sample for the primary analysis was assessed after data collection from the precision of the item-level estimates and the stability of the priority classification under resampling (Section 2.5).

### 2.3. Measurements

Data were collected using a structured, self-report questionnaire. Permission to use each existing instrument was obtained from the original developers. The reliability of each instrument in the present sample is reported in Section 3.2.

#### 2.3.1. Perceived Knowledge of NIV Care

Perceived knowledge was measured with the ventilator nursing knowledge instrument developed by Kim et al. [7]. The original items were derived from the American Association for Respiratory Care clinical practice guideline on the initiation and management of mechanical ventilation and adult nursing texts on ventilator nursing interventions; items relating to invasive ventilation were excluded and only content applicable to non-invasive positive pressure ventilation was retained. In the original study, content validity was established through sequential review by clinical and academic experts (CVI = 1.00). The instrument comprises 11 items covering indications, ventilation principles, device and circuit management, discomfort and complications, interpretation of monitored values, documentation and infection prevention. Participants rated how well they knew each item on a scale from 1 to 10, giving a possible total score of 11–110, with higher scores indicating greater self-rated knowledge. Because participants rated their own level of knowledge rather than answering items scored as correct or incorrect, the construct is referred to throughout as perceived knowledge. For this study, the term “mechanical ventilator” was replaced with “non-invasive ventilation” throughout to match the practice context. Cronbach’s α was 0.95 in the original study.

#### 2.3.2. Self-Efficacy in NIV Care

Self-efficacy was measured with the companion instrument developed by Kim et al. [7] using the same procedure. The 15 items address confidence, self-regulatory efficacy and preference for task difficulty across the assessment, nursing diagnosis and planning, implementation, and evaluation phases of NIV nursing. Each item was rated from 1 to 10, giving a possible total score of 15–150, with higher scores indicating greater self-efficacy. Cronbach’s α was 0.97 in the original study.

#### 2.3.3. Implementation Climate

Implementation climate was measured with the Implementation Climate Scale [10], which comprises 18 items in six subscales of three items each: focus on evidence-based practice, educational support, recognition, rewards, selection for evidence-based practice and selection for openness. Items were rated from 0 (not at all) to 4 (to a very great extent), giving a possible total score of 0–72, with higher scores indicating a more favourable perception. Subscale reliabilities in the original study ranged from 0.81 to 0.91. With the developer’s permission, a Korean version was prepared through forward and back translation by two independent bilingual translators, and the research team compared the back-translation with the original to confirm semantic equivalence. Comprehensibility was then assessed with five ICU nurses. No further psychometric evaluation of the Korean version was undertaken, and its construct validity in this population has not been established. Because the scale measures the climate for evidence-based practice in the unit as a whole rather than for a specific intervention, the item wording was retained unchanged. Scores were analysed as individual nurses’ perceptions of the climate of their own unit and were not aggregated to the unit level (Section 2.5).

#### 2.3.4. Educational Needs for Evidence-Based NIV Care

Educational need was defined as the discrepancy between the perceived importance of a competency required for NIV care and the level at which the nurses rated themselves as currently able to perform it. Existing instruments measure the level of capability attained [6,7] and provide neither the paired ratings of importance and performance required by the Borich needs assessment and the Locus for Focus model nor coverage of guideline-derived competencies at a resolution sufficient to rank them. An instrument was therefore developed for this study; its content validity indices and the internal consistency in the present sample are reported in Section 3.2.

Items were derived from the European Respiratory Society/American Thoracic Society clinical practice guideline on NIV for acute respiratory failure [3], the American Association for Respiratory Care clinical practice guideline on patient–ventilator assessment [20] and the clinical guide for NIV in adults with acute respiratory failure issued by the New South Wales Agency for Clinical Innovation [21]. Indications, contraindications and disease-specific principles of application were drawn from the first source; assessment of ventilator parameters, inspection of the circuit and interface, and the adequacy of humidification from the second; and secretion management, patient–ventilator asynchrony and the organising structure of the domains from the clinical guide. The source of each item is listed in Supplementary Table S1.

These sources were consolidated into six domains: A, indications and pre-application assessment; B, equipment setup and application; C, monitoring; D, complication management; E, treatment failure and weaning; and F, patient/caregiver education and communication, yielding 36 preliminary items. The domains were used to organise the content of the instrument and summarise item-level results and were not conceived as psychometric subscales. Content validity was evaluated by a panel of five experts (one intensivist, one pulmonologist, one ICU nurse with at least ten years of experience, one advanced practice nurse specialising in pulmonology, and one adult nursing professor) using a 4-point relevance scale, with an item-level content validity index (I-CVI) criterion of ≥0.78 for retention [22]. One item below the criterion was deleted, and the wording of nine items was revised in response to panel comments (Supplementary Table S1). The revised wording was reviewed and agreed within the research team and was not returned to the panel for re-rating. Five ICU nurses who were not study participants then completed the instrument, recorded the time taken, and indicated for each item whether the wording was clear and whether it overlapped with another item; completion of this instrument took approximately 10 min, and no items were reworded before it was finalised.

The final instrument comprises 35 items across the six domains (6, 6, 6, 6, 6 and 5 items respectively). For each item, respondents provided two ratings. The first, the required competency level (RCL), a term retained from the Borich model, is a rating of the perceived importance of the competency in ICU NIV nursing practice, from 1 (not important at all) to 5 (very important); it does not represent a normative performance threshold. The second, the present competency level (PCL), is a rating of the extent to which the respondent is currently able to perform the competency, from 1 (not able to perform at all) to 5 (able to perform very well). Because the two ratings are anchored differently, their difference is interpreted as a perceived gap between the importance of a competency and the respondent’s ability to perform it, the quantity that the Borich model weights [13]. The same paired format has been used in previous nursing applications of the method [16,17]. The instrument was developed and administered in Korean; the full instrument is provided as Supplementary File S1.

#### 2.3.5. Participant Characteristics

The demographic and professional characteristics of ICU nurses included sex, age, highest level of education, total clinical experience, current unit, length of intensive care experience, experience of caring for patients receiving NIV, and previous NIV-related education.

### 2.4. Data Collection

Data were collected between June and July 2026 using a self-administered paper questionnaire. After approval by the institutional review board, the researcher visited each unit, explained the purpose and procedures of the study and distributed the questionnaire to nurses who met the eligibility criteria. The researcher held no supervisory or evaluative role in relation to the participants. Nurses who agreed to participate completed the questionnaire during a break in their shift and returned it in a sealed envelope to a collection box on each unit, which was emptied only by the researcher. Unit managers could not identify which nurses had participated or what they had reported.

### 2.5. Data Analysis

Data were analysed using SPSS/WIN 30.0 (IBM Corp., Armonk, NY, USA), with statistical significance set at α = 0.05. Missing item responses were replaced by person-mean imputation, in which the participant’s mean of the remaining items in the same instrument was substituted and rounded to the nearest scale point. This method preserves the participant’s own response level and, with few missing values, has negligible influence on scale means. The number and location of missing responses, and a complete-case check of the affected analyses, are reported in Section 3.2. Distributional assumptions were examined from the skewness and kurtosis of the total scores of each instrument and of the 35 item-level RCL − PCL differences, and from the residuals of the regression models.

All variables were summarised as frequencies, percentages, means and standard deviations. Because the instruments differ in the number of items and in response range, both total scores and item means are reported. Internal consistency was assessed using Cronbach’s α, which was taken as evidence of internal consistency only and not of construct validity.

Differences in perceived knowledge, self-efficacy and implementation climate according to participant characteristics were examined using independent *t*-tests and one-way analysis of variance. Length of intensive care experience was dichotomised at three years, the point at which nurses are generally considered to reach the competent stage of skill acquisition [23]. Because dichotomisation discards information, intensive care experience and total clinical experience were also analysed in years, by Pearson’s correlation and in the multivariable models described below. Levene’s test was used to examine homogeneity of variance; where the assumption was not met, Welch’s test with Games–Howell post hoc comparisons was used, and the Scheffé method was applied otherwise. Effect sizes were reported with 95% confidence intervals (CIs): Cohen’s *d*, calculated with the pooled standard deviation whichever *t*-test was applied, and η^2^, calculated from the ordinary analysis of variance as the ratio of between-group to total sums of squares, with its CI obtained from the non-central *F* distribution. These comparisons addressed secondary aims and were exploratory. Unadjusted *p* values are reported, and the Holm procedure [24] was applied across the 24 comparisons (eight characteristics by three variables) to identify the associations that remained significant after adjustment for multiple testing.

Because participant characteristics were interrelated, in particular unit assignment and experience of NIV care, multiple linear regression was used to examine whether the associations observed in the bivariate comparisons persisted after mutual adjustment. Perceived knowledge, self-efficacy and implementation climate were each regressed on unit (medical ICU as reference), experience of NIV care, previous NIV-related education, intensive care experience and total clinical experience in years, sex and level of education (bachelor’s degree as reference). Age was not entered because of its collinearity with total clinical experience (*r* = 0.92). Multicollinearity was assessed with variance inflation factors, and heteroscedasticity-consistent (HC3) standard errors were computed to check whether inferences depended on the homoscedasticity assumption. Implementation climate was analysed at the individual level; to assess whether aggregation to the unit level would have been justified, the intraclass correlation coefficient ICC(1), the proportion of variance in individual scores attributable to unit membership, was calculated from a one-way analysis of variance of climate scores by unit [25].

Relationships among perceived knowledge, self-efficacy, implementation climate, RCL, PCL and the perceived competency discrepancy were examined using Pearson’s correlation coefficients. The perceived competency discrepancy is an individual-level score obtained by averaging the RCL−PCL difference across the 35 items and is distinct from the item-level educational need derived through the Borich needs assessment and the Locus for Focus model. Because the discrepancy is computed from RCL and PCL, its correlations with these two variables are partly determined by construction and are reported for completeness only. No adjustment for multiple testing was applied to the regression coefficients or to these correlations; both analyses were exploratory, and their unadjusted *p* values are not treated as confirmatory evidence.

Educational priorities were derived in three steps. First, the difference between RCL and PCL was tested for each of the 35 items using paired *t*-tests, with the Wilcoxon signed-rank test as a check for items whose differences were skewed; because 35 tests were performed, a Bonferroni-corrected criterion of *p* < 0.0014 was applied. Second, the Borich needs assessment score was calculated for each item as the sum of the RCL−PCL differences weighted by the mean RCL, divided by the total number of respondents [13]; domain-level scores were obtained as the mean of the item-level Borich scores within each domain. Third, the Locus for Focus model [14] was applied by plotting mean RCL on the X axis and the mean RCL−PCL difference on the Y axis and dividing the plane into four quadrants at the respective grand means, calculated from the unrounded item means. Following the procedure described by Cho [15] and applied in previous nursing studies [16,17], the number of items taken from the top of the Borich ranking was set equal to the number of items falling in the first quadrant, so that the two solutions select the same number of items and neither dominates the intersection; items appearing in both solutions were designated the highest priority and those appearing in only one solution the second priority. Because items lying close to a reference line may change quadrant with small changes in the sample means, the stability of the classification was examined by non-parametric bootstrap [26], implemented in Python 3.11 (Python Software Foundation, Wilmington, DE, USA). Participants were resampled with replacement 5000 times; the grand means, quadrant allocation and Borich ranking were recalculated in each resample; and the proportion of resamples in which each item fell in Quadrant I among the top-ranked Borich items or in either solution was recorded.

### 2.6. Ethical Considerations

The study was approved by the institutional review board of Chosun University Hospital (approval number: CHOSUN IRB 2026-04-024; approval date 6 May 2026 and conducted in accordance with the Declaration of Helsinki. Participants were informed of the purpose and procedures of the study, the voluntary nature of participation, their right to withdraw at any time, the anonymity of their responses and the restriction of data use to research purposes, and written informed consent was obtained; no identifying information was collected. Because reporting one’s own competency and organisational constraints may cause discomfort, participants were informed before completion that the purpose of the survey was to inform educational support and practice improvement rather than evaluate individuals. The questionnaire took approximately 15 min to complete, and a small gift voucher was provided in return. Data were stored securely and will be destroyed in accordance with institutional policy.

## 3. Results

### 3.1. General and NIV-Related Characteristics of Participants

Of the 146 questionnaires distributed, two were excluded because the respondents had transferred to another unit during data collection and two were incomplete, leaving 142 (97.3% of the eligible nurses in the four units) for analysis. Of the 142 participants, 129 (90.8%) were women and 101 (71.1%) held a bachelor’s degree. The mean age was 31.47 ± 5.78 years. Participants worked in the neurological (39, 27.5%), emergency (36, 25.4%), surgical (34, 23.9%) and medical (33, 23.2%) ICUs. Mean total clinical experience was 7.95 ± 5.92 years and mean intensive care experience was 4.13 ± 3.15 years; 61 nurses (43.0%) had less than three years of intensive care experience. Experience of caring for patients receiving NIV was reported by 107 participants (75.4%), whereas 55 (38.7%) had received NIV-related education. Among those who had, the most frequent sources were continuing education programmes (30, 54.5%) and manufacturer-provided sessions (15, 27.3%) (Table 1). Experience of NIV care was unevenly distributed across units: it was reported by 32 of 33 nurses in the medical ICU (97.0%), 30 of 34 in the surgical ICU (88.2%) and 32 of 36 in the emergency ICU (88.9%), but by 13 of 39 in the neurological ICU (33.3%).

### 3.2. Content Validity, Reliability and Data Screening

In the expert review of the 36 preliminary items of the educational needs instrument, I-CVI ranged from 0.60 to 1.00 and S-CVI/Ave was 0.95. The single item with an I-CVI of 0.60 was deleted, and all 35 retained items had an I-CVI of at least 0.80; these indices refer to the preliminary wording of the items (Section 2.3.4). In the study sample, Cronbach’s α was 0.98 for perceived knowledge, 0.99 for self-efficacy, 0.96 for the total Implementation Climate Scale (0.88–0.97 for the six subscales), and 0.99 for both the RCL and the PCL ratings of the educational needs instrument (Table 2). The very high coefficients for the 35-item RCL and PCL ratings reflect highly consistent responding across items and do not imply that the items measure a single construct.

Four item responses were missing (0.02% of all responses): two in the perceived knowledge items, one in a self-efficacy item and one in an implementation climate item, and none in the RCL or PCL ratings. Excluding the affected participants from the analyses of these three instruments changed item means by at most 0.03 and correlation coefficients by at most 0.01 and altered no inferences.

Skewness of the total scores ranged from −1.31 (RCL) to 0.14 and kurtosis from −0.58 to 1.20; regression residuals were approximately symmetric. Item-level RCL−PCL differences were positively skewed where mean discrepancies were small (skewness up to 1.87, kurtosis up to 3.78), so the Wilcoxon signed-rank test was also applied to these comparisons (Section 3.7).

### 3.3. Perceived Knowledge, Self-Efficacy and Implementation Climate

The item mean for perceived knowledge was 5.47 ± 2.05 on a scale of 1 to 10 and that for self-efficacy was 5.53 ± 2.20; total scores ranged from 11 to 97 and from 15 to 138 respectively. The item mean for implementation climate was 1.81 ± 0.78 on a scale of 0 to 4. Among the six subscales, focus on evidence-based practice was rated highest (2.72 ± 0.71), whereas rewards for evidence-based practice (1.14 ± 1.03) and selection for evidence-based practice (1.51 ± 1.05) were rated lowest. Across the 35 educational content items, the mean importance rating (RCL) was 3.94 ± 1.01 and the mean self-rated present competency level (PCL) was 3.25 ± 0.96, giving a mean perceived competency discrepancy of 0.70 ± 0.82 (Table 2).

### 3.4. Differences in Perceived Knowledge, Self-Efficacy and Implementation Climate According to Participant Characteristics

Perceived knowledge and self-efficacy differed by current unit (Welch’s *F* = 7.93 and 11.45, both *p* < 0.001; η^2^ = 0.199 and 0.258). Nurses in the neurological ICU scored lower than nurses in each of the other three units (Games–Howell, all *p* ≤ 0.001), and the other three units did not differ from one another. Both variables were higher among nurses who had cared for patients receiving NIV (*d* = 2.30, 95% CI 1.84–2.77, and *d* = 2.06, 95% CI 1.61–2.51) and those who had received NIV-related education (*d* = 0.93 and 0.89; all *p* < 0.001). Total clinical experience was associated with both variables (*p* = 0.049 and 0.025; η^2^ = 0.055 and 0.065), but in Scheffé comparisons only the difference in self-efficacy between nurses with ten or more years and those with less than three years was significant (*p* = 0.033). Age was associated with self-efficacy only (*p* = 0.026), and no pairwise comparison was significant. Sex, level of education and intensive care experience dichotomised at three years were not associated with either variable (Table 3). When analysed in years, intensive care experience correlated weakly with perceived knowledge (*r* = 0.17, *p* = 0.039) and self-efficacy (*r* = 0.19, *p* = 0.023), and total clinical experience correlated with self-efficacy (*r* = 0.21, *p* = 0.011) but not significantly with perceived knowledge (*r* = 0.16, *p* = 0.060).

Implementation climate was rated higher by nurses aged 20–29 years than by those aged 40 years or older (Scheffé *p* = 0.030), nurses with a bachelor’s degree versus those with a master’s degree or higher (*p* = 0.036), nurses with less than three years of total clinical experience versus those with ten or more (*p* = 0.006; η^2^ = 0.091), and nurses with less than three years of intensive care experience (*d* = 0.40, *p* = 0.020). Measured in years, intensive care experience and total clinical experience were negatively correlated with implementation climate (*r* = −0.19 and −0.35). Implementation climate did not differ by current unit, sex, experience of NIV care or previous NIV-related education (Table 3).

After Holm adjustment across the 24 comparisons, the differences by unit, experience of NIV care and previous NIV-related education in perceived knowledge and self-efficacy remained significant (adjusted *p* ≤ 0.002). The associations of age, education, total clinical experience and intensive care experience with any of the three variables did not remain significant (smallest adjusted *p* = 0.075).

### 3.5. Multivariable Analysis of Participant Characteristics

In the multiple regression models (Table 4), experience of NIV care (β = 0.60 for perceived knowledge and 0.47 for self-efficacy) and previous NIV-related education (β = 0.20 and 0.21) remained associated with both variables after adjustment for unit, length of experience, sex and education. Once experience of NIV care was included, the neurological ICU did not differ from the medical ICU on either variable (B = 0.00 and −0.71; 95% CIs included zero), and neither intensive care experience nor total clinical experience in years was associated with either variable. The models explained 52.0% and 49.0% of the variance (adjusted *R*^2^). For implementation climate, only total clinical experience remained associated with the score after adjustment (β = −0.27), and the model explained 10.4% of the variance. The ICC(1) for implementation climate by unit was −0.004 (F = 0.84, *p* = 0.473), compared with 0.23 for perceived knowledge and 0.30 for self-efficacy.

### 3.6. Correlation Among the Study Variables

Perceived knowledge and self-efficacy were very highly correlated (*r* = 0.949, 95% CI 0.930–0.963, *p* < 0.001). Both were more strongly related to the present competency level (*r* = 0.783 and *r* = 0.829, both *p* < 0.001) than to the importance rating (RCL) (*r* = 0.592 and *r* = 0.596, both *p* < 0.001) and both showed small negative correlations with the perceived competency discrepancy (*r* = −0.187, *p* = 0.026 and *r* = −0.236, *p* = 0.005), which follow in part from their strong correlation with PCL, a component of the discrepancy. Implementation climate was not significantly correlated with any of these variables (*r* = −0.102 to 0.097, all *p* > 0.05) (Table 5).

### 3.7. Educational Needs and Priorities for NIV Nursing Care

Importance ratings exceeded self-rated present competency for all 35 items (paired t = 4.70–10.66, all *p* < 0.001, below the Bonferroni-corrected criterion; Wilcoxon signed-rank tests, all *p* < 0.001). The standardised mean difference ranged from 0.39 (item 21) to 0.89 (item 16). The largest discrepancies were observed for item 7 (1.01), item 27 (0.99), item 4 (0.97) and item 16 (0.96), and the smallest for items 18 and 21 (0.36 each). At the domain level, the mean Borich score was highest in Domain E (treatment failure and weaning; 3.07), followed by Domain B (equipment setup and application; 2.93), and lowest in Domain D (complication management; 2.33).

**Figure 1:**
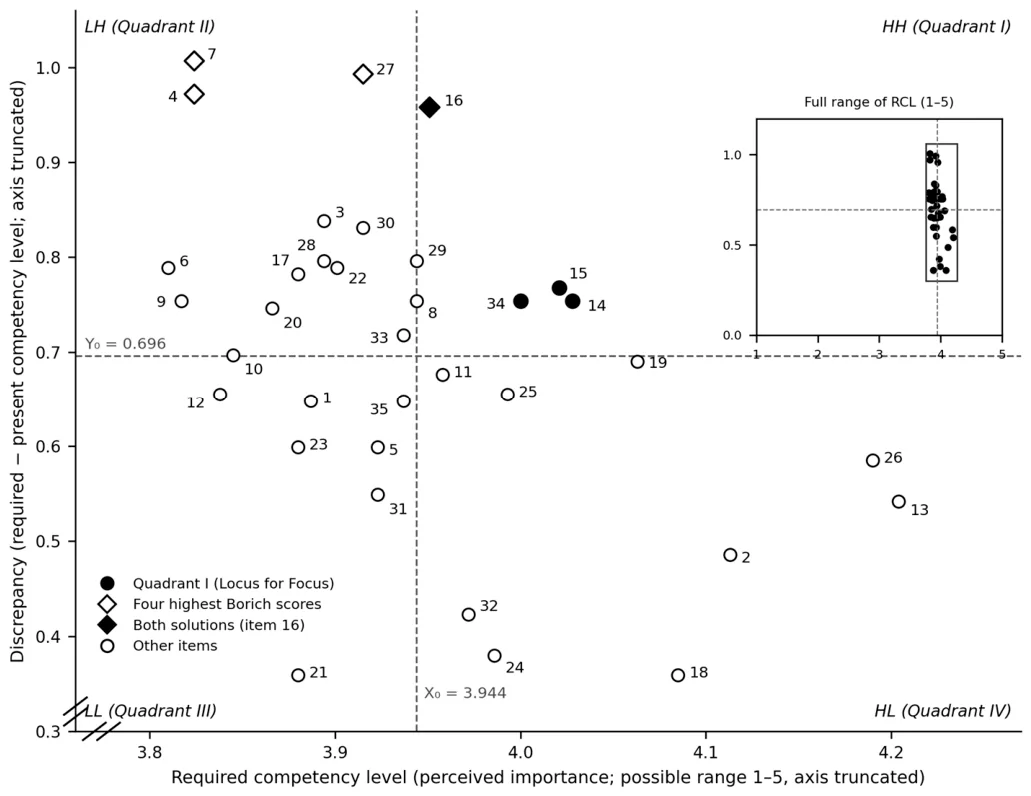
The Locus for Focus model for educational needs in evidence-based NIV care. Note: NIV = non-invasive ventilation. Numbers denote items as listed in Table 6. The reference lines are the grand means of the required competency level (perceived importance; X_0_ = 3.944) and of the required−present difference (Y_0_ = 0.696). Axes are truncated to the observed range of item means (break marks); the inset shows the full 1–5 range. Filled circles: items in Quadrant I; open diamonds: the four highest Borich scores; filled diamond: item 16, selected by both methods.

Weighting by the importance rating altered the ranking. The four highest Borich scores were obtained for item 27 (3.89), item 7 (3.85), item 16 (3.78) and item 4 (3.72), whereas four items fell in Quadrant I of the Locus for Focus model, with reference lines drawn at the grand mean of the importance rating (X_0_ = 3.944) and the discrepancy (Y_0_ = 0.696): items 14, 15, 16 and 34 (Figure 1). The two solutions agreed on one item only. Item 16 appeared in both and was designated the highest priority, and items 4, 7, 14, 15, 27 and 34, each selected by one method, were designated the second priority (Table 6). The three items ranked first, second and fourth by Borich score (27, 7 and 4) lay in Quadrant II because their importance ratings (3.82–3.92) were below the grand mean despite the largest discrepancies in the instrument, whereas the three Quadrant I items not selected by Borich score (14, 15 and 34) combined above-average importance with discrepancies of 0.75–0.77, close to the reference line.

Several items lay close to the importance reference line: item 16 (3.951) was 0.007 above it, items 8 and 29 (3.944) were fractionally below it, and item 27 (3.915) was 0.029 below it. In the bootstrap analysis (Supplementary Table S2), items 14, 15 and 34 fell in Quadrant I in 90.1%, 91.9% and 90.5% of resamples, item 16 in 59.5%, items 29 and 8 in 50.0% and 46.9%, and item 27 in 19.9%. Items 27, 7, 16 and 4 were among the top-ranked Borich items in 99.0%, 98.3%, 97.9% and 96.6% of resamples, and no other item exceeded 47.5% (items 3 and 30). The number of items in Quadrant I varied from 1 to 13 across resamples (median 7). Each of the seven priority items was selected by at least one method in 90.1–99.0% of resamples, whereas the next most frequently selected items (29, 30, 3 and 8) were selected in 49.3–55.6%. The composition of the selected set varied: the number of items selected by at least one method ranged from 2 to 20 (median 11), all seven items were included together in 72.8% of resamples, and the identical seven-item set was reproduced in 0.5% of resamples. The distinction between the first and second priority rested on the position of item 16 above the importance reference line, which was reproduced in 59.5% of resamples; item 16 was selected by both methods in 59.4%.

## 4. Discussion

This survey of 142 ICU nurses prioritised 35 guideline-derived competencies for NIV care by self-perceived educational need, and examined perceived knowledge, self-efficacy and implementation climate for evidence-based practice as secondary outcomes. Nurses rated their knowledge and self-efficacy near the midpoint of each scale (item means 5.47 and 5.53 of 10). These levels are higher than the preparation reported among nurses without NIV-specific training [5], although the two measures are not directly comparable, and brief structured programmes have improved both knowledge and self-efficacy in intervention studies [7,18,19]. The correlation between perceived knowledge and self-efficacy (*r* = 0.949) was higher than would be expected of two distinct constructs. Both instruments are self-appraisals completed within the same questionnaire, with the same 10-point response format and items that refer to the same phases of NIV nursing. The correlation may reflect common method variance [27], overlap in item content, or both. In either case, the two scales showed limited discriminant validity in this sample, and differences between their associations with other variables should not be interpreted. Whether knowledge and confidence diverge in this population could be examined with an objective knowledge test or observed or simulation-based assessment of performance.

Both variables were related more closely to exposure to NIV than to length of experience. Nurses in the neurological ICU scored lower than those in the medical, surgical and emergency units (η^2^ = 0.199 and 0.258), and, although the four units share the same NIV protocol, only one-third of them had ever cared for a patient receiving NIV, compared with 88–97% in the other units. Nurses who had cared for such patients, or who had received NIV-specific education, scored higher (*d* = 2.30 and 2.06; *d* = 0.93 and 0.89), and these effects were larger than those of total clinical experience (η^2^ = 0.055 and 0.065), which did not survive adjustment for multiple testing. In the regression models, experience of NIV care and previous NIV-related education remained associated with both variables, the neurological ICU no longer differed from the other units, and neither intensive care experience nor total clinical experience in years was associated with either variable. The attenuation of the unit difference after adjustment is consistent with a contribution of the uneven distribution of NIV exposure across units, but it does not show that exposure accounts for the difference. Because the design is cross-sectional, these associations do not show that exposure produces capability. Nurses who feel more capable may be assigned to, or seek, NIV patients, and unmeasured differences in case mix and educational opportunity between units cannot be excluded. Leonardsen et al. [28] described the first application of an NIV mask as the most demanding moment in this area of practice, requiring a deliberate balance between patient safety and autonomy, and Sørensen et al. [29] characterised NIV decision-making as practical wisdom accumulated case by case. The three-year threshold conventionally used to mark the competent stage of skill acquisition [23] did not on its own distinguish nurses’ self-appraised readiness for NIV care, and the weak correlations with experience measured in years (*r* = 0.16–0.21) indicate that this conclusion does not depend on the chosen cut-point. If this pattern is confirmed elsewhere, unit assignment and case exposure, as well as tenure, would need to be considered when educational resources are allocated.

Implementation climate was rated below the midpoint of the scale (item mean 1.81 of 4), with rewards for evidence-based practice rated lowest, as in the original validation study [10]. It was not significantly correlated with perceived knowledge, self-efficacy or any index of educational need. It was rated more favourably by less experienced nurses than by senior colleagues (η^2^ = 0.091); the association with total clinical experience persisted in the adjusted model but not after adjustment for multiple testing in the bivariate analysis. The absence of a correlation is not evidence that individual capability and organisational climate are independent, and the inference is limited in other ways. The scale measures the climate for evidence-based practice in general, not the conditions surrounding NIV care, so a relationship confined to the NIV-specific environment would not have been detected. Nurses were nested within only four units, and with an ICC(1) of −0.004 there was no between-unit variance in climate perceptions to analyse; the scores represent individual perceptions and not a shared unit property. The single-centre design also provides no variation in hospital-level policy. In this sample, nurses’ self-appraised capability in NIV care and their perception of the unit’s support for evidence-based practice were not correlated, which accords with reports that climate has a direct effect on evidence-based practice behaviour [11] and that individual characteristics account for little of the variance in how climate is perceived [12]. Oral hygiene during NIV (item 24) shows why the distinction matters for needs assessment. It had one of the smallest discrepancies in the instrument (0.38, ranked 33rd), yet coverage of the mouth by the interface has been identified as a reason for omitting oral care in practice, independently of nurses’ knowledge of its importance [30].

The highest mean Borich score was obtained in Domain E (treatment failure and weaning; 3.07), and four of the seven priority candidates fell in this domain and in Domain C (monitoring): ventilator-waveform monitoring (item 16), evaluation of treatment response within 1–4 h (item 14), interpretation of arterial blood gas results for setting adjustment (item 15) and patient–ventilator asynchrony (item 27). The clinical consequences of these competencies were not measured in this study. In the wider literature, the benefit of NIV in preventing reintubation is confined to patients identified in advance as being at high risk [31,32], failure of primary support is detectable through indices measured at fixed early intervals [33,34], and delayed escalation is associated with worse outcomes [4]. It is possible, although the present data cannot show it, that nurses’ perceived educational need corresponded to the competencies in which misjudgement carries the greatest clinical consequence rather than to those performed most frequently. Waveform interpretation, blood gas-guided titration and the recognition of asynchrony require clinical reasoning applied to changing patient data. This study did not compare educational methods, and no conclusion about the relative effectiveness of didactic teaching, simulation or case-based discussion can be drawn from it. In the educational literature, competencies of this kind are taught with methods that require learners to interpret physiological data and decide on a course of action within a limited time; whether such methods improve NIV nursing has not been tested.

The item-level results showed two contrasts. Interpreting blood gas criteria to decide whether to initiate NIV (item 2) ranked 31st in Borich score, whereas interpreting the same results to adjust settings once NIV is underway (item 15) ranked 9th and fell in Quadrant I of the Locus for Focus model. This study did not examine the allocation of responsibilities between nurses and physicians, and the initiation criteria for NIV differ by cause of respiratory failure, local protocol and team structure [3,4,35]. The contrast may partly reflect the difference between initiation, which, in most settings, follows protocolised criteria and a medical prescription, and the nurse’s continuing role after initiation, which is to monitor the response, recognise when it is inadequate and communicate the need for adjustment. Item 27 is worded accordingly as requesting an adjustment and not making it.

Conversely, items addressing interface selection, fitting and leak management (items 9, 10 and 20) and direct communication with patients and families (items 31–33) did not rank highly, and the lowest mean Borich score was obtained in Domain D (complication management; 2.33), which covers air leak, skin damage and patient discomfort. This finding diverges from evidence that interface management is among the most labour-intensive aspects of NIV nursing [36], from safety-incident data showing that most reported NIV-related adverse events involve interface-related pressure injury [21] and from descriptions of pressure injury as a persistent grey area of NIV nursing [37]. Sustained quality improvement attention to device-related pressure injury [38] may have raised nurses’ confidence in this domain, but low reported need may also reflect limited awareness of a deficit in routine practice. The low priority assigned to these domains therefore does not indicate that further education is unnecessary. Needs assessment based on self-report is better used to inform the content of education than to determine it, and domains in which reported need and recorded adverse events diverge should be reviewed against practice data before content is finalised.

The two prioritisation methods agreed on a single item. The Borich score rewards a large discrepancy weighted by importance, so items 27, 7 and 4, with the largest discrepancies but below-average importance ratings, ranked highest, whereas the Locus for Focus model requires above-average importance and selected items 14, 15 and 34, whose discrepancies were moderate. The disagreement shows that, in this sample, the competencies nurses felt least able to perform were not those they rated as most important. It also shows how sensitive the quadrant classification is to small differences. All item-level discrepancies met the Bonferroni-corrected criterion, but the sample was not large enough to separate items lying close to a reference line. Fourteen of the 35 items had importance ratings within 0.05 of the importance reference line, a distance smaller than the 95% CI half-width of the item means (approximately 0.18), and item 16 fell in Quadrant I in only 59.5% of bootstrap resamples. Each of the seven items was selected by at least one method in at least 90% of resamples, but the same seven-item set was reproduced in fewer than 1% of resamples and the number of items selected varied widely (median 11), so the composition and ranking of the priority set were not stable. The designation of item 16 as the single highest priority, and the exclusion of items 8, 29 and 3, which were selected in about half of the resamples, should not be read as definitive. For this reason, we describe the seven items as priority candidates. Previous applications of the combined method [16,17] have not reported the stability of their classifications; doing so would allow the precision of priority sets to be compared across studies.

### 4.1. Implications for Nursing Education

Needs assessment identifies which of the competencies required in practice should be prioritised when educational resources are limited. Although importance exceeded self-rated present competency for all 35 guideline-derived competencies, the two criteria in combination narrowed them to seven priority candidates, a set that neither criterion identified on its own. Four of these seven address bedside physiological judgement: interpretation of ventilator waveforms, structured evaluation of treatment response during the first hours of NIV, titration guided by arterial blood gas results, and recognition of patient–ventilator asynchrony. The remaining two, contraindications to NIV and differences between NIV modes, concern the knowledge base on which the decision to initiate NIV rests, whereas the seventh, structured reporting to the care team of NIV-related changes in patient status, links the recognition of deterioration to its communication. These findings support additional emphasis on the seven competencies within a comprehensive NIV curriculum. They do not support omitting lower-ranked competencies: interface management and patient and caregiver education should be retained wherever guideline recommendations, adverse-event data or observed practice indicate continued safety relevance, and self-reported priority should supplement rather than replace such requirements when content is selected.

Other implications come from the secondary analyses and the wider literature, not from the needs assessment itself. Once NIV case exposure and NIV-specific education were taken into account, tenure showed no independent association with perceived knowledge or self-efficacy, so allocating educational resources by unit as well as by seniority appears reasonable. Structured practice such as simulation is one means by which nurses in units where NIV is used infrequently could gain exposure, although whether it improves performance in this group has not been tested. Implementation climate was rated lowest for rewards and selection for evidence-based practice. This study found no association between climate and self-appraised capability, so the present data do not show that improving the climate would improve NIV care. In the implementation literature, however, individual education seldom changes practice where the organisational conditions for the practice are weak [9,11], and recognition and reward for evidence-based practice deserve attention in their own right. Needs assessment can inform what to teach and indicate where educational resources may be needed most, decisions that an evaluation conducted after implementation cannot address.

### 4.2. Limitations

This study has several limitations. All variables were self-reported. Knowledge and self-efficacy were measured by self-appraisal rather than by objective testing or observed performance, and the two measures showed limited discriminant validity in this sample, so the findings describe self-perceived rather than demonstrated capability. The same applies to the educational priorities, which rest on nurses’ own ratings of the importance of each competency and their present ability to perform it. These two ratings have different anchors, and their difference is a perceived gap, not a measured performance deficit. The Borich model is designed for ratings of this kind, but treating the difference as an educational need assumes that nurses can appraise both accurately. Needs identified by one group have been shown to diverge from those identified by another [39], so the priorities reported here should be read as nurses’ appraisal of their own learning needs rather than as an externally validated account of them. Because all variables were reported by the same participants at a single time point, common method variance may also have inflated the associations observed [27], and the cross-sectional design does not allow any inference about the direction of the association between NIV exposure and self-appraised capability.

The educational needs instrument was developed for this study. Its content validity was established by a panel of five experts, which is sufficient for a preliminary assessment but small, and its evaluation was otherwise limited to internal consistency. The revised wording of nine items was agreed within the research team and was not re-rated by the panel, so the content validity of the final wording was not fully established. Construct validity, factor structure and test–retest reliability were not examined, and the six domains were used to organise content and were not validated as subscales. The Korean version of the Implementation Climate Scale was prepared by translation and back-translation and tested for comprehensibility, but its psychometric properties were not formally evaluated. Implementation climate was analysed as individual perceptions. With four units and no detectable between-unit variance, the study could not assess climate as a unit-level property, and the general evidence-based practice focus of the scale may have limited its sensitivity to conditions specific to NIV care.

The priority classification was sensitive to small differences around the reference lines of the Locus for Focus model. Item 16, the only item selected by both methods, was located in Quadrant I in 59.5% of bootstrap resamples, several items excluded from the priority set were selected in about half of the resamples, and the same seven-item set was reproduced in fewer than 1% of resamples. The individual candidates were selected consistently, but the composition and ranking of the set were not, and the priorities are best regarded as candidates, not as a definitive set. Comparisons across participant characteristics were exploratory. Twenty-four bivariate tests and 35 paired tests were performed, and although the Holm and Bonferroni procedures were applied, the associations that did not survive adjustment, including those with total clinical experience and age, may be chance findings. The regression coefficients and the correlations in Table 5 were not adjusted for multiple testing, and their unadjusted *p* value should not be read as confirmatory evidence. Some subgroups were small (13 men, 16 nurses aged 40 years or older), and the corresponding estimates are imprecise. The regression models adjusted for measured characteristics only and cannot separate the effects of unit assignment, case exposure, prior education and experience from unmeasured differences between units. Intensive care experience was dichotomised at three years in the group comparisons; the continuous analyses gave the same conclusion, but other cut-points were not examined. Participants were recruited from the ICUs of a single tertiary hospital, and the priorities identified reflect the case mix and educational provision of that setting.

## 5. Conclusions

Importance ratings exceeded self-rated present competency for every one of the 35 guideline-derived competencies for NIV care, and the Borich needs assessment and the Locus for Focus model in combination identified seven priority candidates, of which monitoring of ventilator waveforms was the only competency selected by both methods. The candidates concentrated on monitoring and the recognition of treatment failure, competencies that involve interpretation of changing physiological data, although the consequences of deficits in these competencies for patients were not examined. The priorities are self-perceived, derived from a newly developed instrument and were sensitive to small differences at the classification thresholds. They can inform the emphasis given to content within a comprehensive NIV curriculum but should not determine its scope. Self-appraised knowledge and self-efficacy were associated with direct exposure to NIV and NIV-specific education and showed no independent association with length of intensive care experience after adjustment. Unit assignment, as well as tenure, may need to be considered when education is provided, and structured exposure such as simulation is one option for nurses in units where NIV is used infrequently, although its effect has not been evaluated. Implementation climate was not correlated with self-appraised capability in this sample, and organisational support for evidence-based practice is a consideration from the implementation literature, not a finding of this study. Future work should verify these priorities against observed performance, examine the construct validity of the instrument, and test prospectively whether a curriculum emphasising the priority competencies improves clinical decision-making and outcomes for patients receiving NIV.

## Figures and Tables

**Table 1:** General and NIV-related characteristics of participants (*N* = 142).

Characteristics	Categories	*n* (%)
Sex	Female	129 (90.8)
	Male	13 (9.2)
Age (years)	20–29	60 (42.3)
	30–39	66 (46.5)
	≥40	16 (11.3)
	M ± SD	31.47 ± 5.78
Education	Diploma	17 (12.0)
	Bachelor	101 (71.1)
	Master or above	24 (16.9)
Current unit	Medical ICU	33 (23.2)
	Surgical ICU	34 (23.9)
	Neurological ICU	39 (27.5)
	Emergency ICU	36 (25.4)
Total clinical experience (years)	<3	29 (20.4)
	3–<5	25 (17.6)
	5–<10	53 (37.3)
	≥10	35 (24.6)
	M ± SD	7.95 ± 5.92
ICU experience (years)	<3	61 (43.0)
	≥3	81 (57.0)
	M ± SD	4.13 ± 3.15
Experience of caring for patients receiving NIV	Yes	107 (75.4)
	No	35 (24.6)
Previous NIV-related education	Yes	55 (38.7)
	No	87 (61.3)
Type of NIV education received (*n* = 55) ^a^	Continuing education	30 (54.5)
	Manufacturer-provided	15 (27.3)
	Online course	4 (7.3)
	Conference	2 (3.6)
	Other	7 (12.7)

Note: ICU = intensive care unit; NIV = non-invasive ventilation. ^a^ = Multiple responses were permitted; percentages are of the 55 nurses who reported previous NIV-related education.

**Table 2:** Perceived knowledge, self-efficacy, implementation climate and competency levels (*N* = 142).

Variables	Total Score M ± SD	Observed Range	Item Mean M ± SD	Cronbach’s α
Perceived knowledge (11 items, 1–10)	60.15 ± 22.53	11–97	5.47 ± 2.05	0.98
Self-efficacy (15 items, 1–10)	82.97 ± 33.00	15–138	5.53 ± 2.20	0.99
Implementation climate, total (18 items, 0–4)	32.66 ± 14.00	5–72	1.81 ± 0.78	0.96
Focus on EBP (3 items)	8.15 ± 2.13	3–12	2.72 ± 0.71	0.88
Educational support (3 items)	5.44 ± 2.59	0–12	1.81 ± 0.86	0.92
Recognition (3 items)	5.87 ± 2.76	0–12	1.96 ± 0.92	0.89
Rewards (3 items)	3.43 ± 3.08	0–12	1.14 ± 1.03	0.96
Selection for EBP (3 items)	4.54 ± 3.14	0–12	1.51 ± 1.05	0.97
Selection for openness (3 items)	5.24 ± 3.02	0–12	1.75 ± 1.01	0.97
Required competency level (perceived importance) (35 items, 1–5)	138.04 ± 35.47	35–175	3.94 ± 1.01	0.99
Present competency level (self-rated) (35 items, 1–5)	113.70 ± 33.68	35–171	3.25 ± 0.96	0.99
Perceived competency discrepancy	–	–	0.70 ± 0.82	–

Note: EBP = evidence-based practice Possible total score ranges were 11–110 for perceived knowledge, 15–150 for self-efficacy, 0–72 for implementation climate and 35–175 for the required and present competency levels. The RCL is a rating of the perceived importance of each competency and the PCL a self-rating of present ability to perform it. The perceived competency discrepancy is the mean of the item-level differences between the required and the present competency level.

**Table 3:** Differences in perceived knowledge, self-efficacy and implementation climate according to participant characteristics (*N* = 142).

			Perceived Knowledge			Self-Efficacy			Implementation Climate		
Characteristics	Categories	*n*	M ± SD	*t* or *F* (*p*)	Effect Size (95% CI)	M ± SD	*t* or *F* (*p*)	Effect Size (95% CI)	M ± SD	*t* or *F* (*p*)	Effect Size (95% CI)
Sex	Female	129	5.56 ± 2.03	1.68 (0.096)	Δ 0.99 (−0.18, 2.16) *d* 0.49 (−0.09, 1.06)	5.62 ± 2.18	1.52 (0.131)	Δ 0.97 (−0.29, 2.23) *d* 0.44 (−0.13, 1.02)	1.78 ± 0.77	−1.87 (0.063)	Δ −0.42 (−0.86, 0.02) *d* −0.55 (−1.12, 0.03)
	Male	13	4.57 ± 2.11			4.65 ± 2.29			2.20 ± 0.82		
Age (years)	20–29	60	5.05 ± 2.05	2.45 (0.090)	η^2^ 0.034 (0.000, 0.102)	5.02 ± 2.21	3.73 (0.026)	η^2^ 0.051 (0.000, 0.128)	1.98 ± 0.82 ^x^	3.91 (0.022)	η^2^ 0.053 (0.000, 0.132)
	30–39	66	5.71 ± 1.94			5.76 ± 2.05			1.76 ± 0.71 ^xy^		
	≥40	16	6.06 ± 2.27			6.52 ± 2.42			1.41 ± 0.71 ^y^		
Education	Diploma	17	5.78 ± 1.94	0.82 (0.442)	η^2^ 0.012 (0.000, 0.059)	5.94 ± 2.04	2.00 (0.140)	η^2^ 0.028 (0.000, 0.092)	1.59 ± 0.84 ^xy^	4.26 (0.016)	η^2^ 0.058 (0.002, 0.138)
	Bachelor	101	5.33 ± 2.11			5.30 ± 2.22			1.93 ± 0.76 ^x^		
	Master or above	24	5.84 ± 1.85			6.21 ± 2.10			1.48 ± 0.71 ^y^		
Current unit	Medical ICU	33	5.96 ± 1.06 ^x^	7.93 ^a^ (<0.001) †	η^2^ 0.199 (0.081, 0.299)	6.16 ± 1.16 ^x^	11.45 ^a^ (<0.001) †	η^2^ 0.258 (0.130, 0.358)	1.68 ± 0.75	0.84 (0.473)	η^2^ 0.018 (0.000, 0.063)
	Surgical ICU	34	5.98 ± 1.82 ^x^			6.29 ± 1.93 ^x^			1.83 ± 0.81		
	Neurological ICU	39	3.99 ± 2.41 ^y^			3.72 ± 2.44 ^y^			1.96 ± 0.90		
	Emergency ICU	36	6.13 ± 1.75 ^x^			6.19 ± 1.80 ^x^			1.77 ± 0.61		
Total clinical experience (years)	<3	29	4.58 ± 2.09	2.68 (0.049)	η^2^ 0.055 (0.000, 0.127)	4.60 ± 2.24 ^x^	3.21 (0.025)	η^2^ 0.065 (0.000, 0.141)	2.19 ± 0.83 ^x^	4.61 (0.004)	η^2^ 0.091 (0.011, 0.176)
	3–<5	25	5.44 ± 1.58			5.29 ± 1.85 ^xy^			1.92 ± 0.80 ^xy^		
	5–<10	53	5.67 ± 2.18			5.70 ± 2.24 ^xy^			1.77 ± 0.69 ^xy^		
	≥10	35	5.93 ± 1.94			6.22 ± 2.12 ^y^			1.50 ± 0.73 ^y^		
ICU experience (years)	<3	61	5.11 ± 2.12	−1.80 (0.074)	Δ −0.62 (−1.30, 0.06) *d* −0.31 (−0.64, 0.03)	5.16 ± 2.27	−1.78 (0.078)	Δ −0.66 (−1.39, 0.07) *d* −0.30 (−0.64, 0.03)	1.99 ± 0.78	2.36 (0.020)	Δ 0.31 (0.05, 0.56) *d* 0.40 (0.06, 0.74)
	≥3	81	5.74 ± 1.97			5.81 ± 2.11			1.68 ± 0.75		
Experience of NIV care	Yes	107	6.29 ± 1.30	9.93 ^a^ (<0.001) †	Δ 3.35 (2.67, 4.03) *d* 2.30 (1.84, 2.77)	6.37 ± 1.45	8.76 ^a^ (<0.001) †	Δ 3.39 (2.61, 4.17) *d* 2.06 (1.61, 2.51)	1.76 ± 0.75	−1.59 (0.114)	Δ −0.24 (−0.54, 0.06) *d* −0.31 (−0.69, 0.07)
	No	35	2.95 ± 1.85			2.98 ± 2.13			2.00 ± 0.83		
Previous NIV- related education	Yes	55	6.54 ± 1.34	5.98 ^a^ (<0.001) †	Δ 1.74 (1.17, 2.32) *d* 0.93 (0.58, 1.29)	6.64 ± 1.52	5.66 ^a^ (<0.001) †	Δ 1.81 (1.17, 2.44) *d* 0.89 (0.54, 1.25)	1.84 ± 0.68	0.28 (0.782)	Δ 0.04 (−0.23, 0.30) *d* 0.05 (−0.29, 0.39)
	No	87	4.79 ± 2.14			4.83 ± 2.28			1.80 ± 0.84		

Note: CI = confidence interval; ICU = intensive care unit; NIV = non-invasive ventilation. Values are item means ± SD. Δ = mean difference between the two categories; *d* = Cohen’s *d* (pooled SD); η^2^ from analysis of variance; 95% CIs in parentheses. ^a^ Welch’s test with Games–Howell post hoc comparisons (unequal variances); Scheffé post hoc comparisons otherwise. ^x, y^ Within a characteristic, means not sharing a letter differ (*p* < 0.05). † Significant after Holm adjustment across the 24 comparisons.

**Table 4:** Multiple linear regression of perceived knowledge, self-efficacy and implementation climate on participant characteristics (*N* = 142).

Predictor	Perceived Knowledge			Self-Efficacy			Implementation Climate		
	*B* (95% CI)	β	*p*	*B* (95% CI)	β	*p*	*B* (95% CI)	β	*p*
Surgical ICU (ref. medical ICU)	0.30 (−0.42, 1.01)	0.06	0.412	0.33 (−0.46, 1.12)	0.06	0.412	0.24 (−0.13, 0.61)	0.13	0.209
Neurological ICU	0.00 (−0.82, 0.82)	0.00	0.999	−0.71 (−1.62, 0.19)	−0.15	0.122	0.23 (−0.19, 0.65)	0.13	0.284
Emergency ICU	0.44 (−0.26, 1.15)	0.09	0.217	0.28 (−0.51, 1.06)	0.05	0.487	0.13 (−0.23, 0.50)	0.07	0.476
Experience of NIV care (yes)	2.83 (2.09, 3.58)	0.60	<0.001	2.38 (1.55, 3.21)	0.47	<0.001	−0.07 (−0.46, 0.32)	−0.04	0.715
Previous NIV-related education (yes)	0.85 (0.32, 1.37)	0.20	0.002	0.93 (0.35, 1.51)	0.21	0.002	0.12 (−0.15, 0.40)	0.08	0.367
ICU experience (years)	0.00 (−0.08, 0.09)	0.00	0.973	0.01 (−0.09, 0.10)	0.01	0.885	−0.03 (−0.07, 0.02)	−0.11	0.236
Total clinical experience (years)	0.01 (−0.05, 0.06)	0.02	0.815	0.02 (−0.04, 0.08)	0.06	0.491	−0.04 (−0.06, −0.01)	−0.27	0.014
Female	0.73 (−0.13, 1.58)	0.10	0.094	0.81 (−0.13, 1.76)	0.11	0.091	−0.35 (−0.79, 0.10)	−0.13	0.123
Diploma (ref. bachelor)	0.00 (−0.80, 0.80)	0.00	0.995	0.06 (−0.83, 0.94)	0.01	0.897	−0.15 (−0.56, 0.27)	−0.06	0.486
Master or above (ref. bachelor)	0.18 (−0.63, 0.99)	0.03	0.668	0.33 (−0.56, 1.23)	0.06	0.463	−0.05 (−0.47, 0.37)	−0.02	0.816
*R*^2^ (adjusted *R*^2^); *F*(10, 131) (*p*)	0.554 (0.520); 16.29 (<0.001)			0.526 (0.490); 14.54 (<0.001)			0.168 (0.104); 2.64 (0.006)		

Note: *B* = unstandardised coefficient; β = standardised coefficient; CI = confidence interval; ICU = intensive care unit; NIV = non-invasive ventilation. Outcomes are item means. Reference categories: medical ICU, no NIV care experience, no NIV education, male, bachelor’s degree. Age was omitted (*r* = 0.92 with total clinical experience). Variance inflation factors ≤ 2.40; conclusions unchanged with HC3 standard errors.

**Table 5:** Correlations among perceived knowledge, self-efficacy, implementation climate and competency levels (*N* = 142).

Variables	1	2	3	4	5	6
1. Perceived knowledge	1					
2. Self-efficacy	0.949 ***	1				
3. Implementation climate	0.043	0.057	1			
4. Required competency level	0.592 ***	0.596 ***	0.010	1		
5. Present competency level	0.783 ***	0.829 ***	0.097	0.656 ***	1	
6. Perceived competency discrepancy	−0.187 *	−0.236 **	−0.102	0.466 ***	−0.363 ***	1

Note: * *p* < 0.05, ** *p* < 0.01, *** *p* < 0.001.

**Table 6:** Educational needs for evidence-based NIV care by item: paired *t*-tests, Borich needs assessment, the Locus for Focus model and bootstrap stability (*N* = 142).

Domain/Item	RCL M ± SD	PCL M ± SD	RCL − PCL	*t*	Borich Score	Borich Rank	LFF Quadrant	Priority	Bootstrap (%) ^a^ Q I/Top-*k*
*A. Indications and pre-application assessment*									
1. Identify clinical situations that should prompt consideration of NIV	3.89 ± 1.08	3.24 ± 1.08	0.65	8.03	2.52	24	III		5/0
2. Interpret arterial blood gas criteria for determining NIV Initiation	4.11 ± 1.11	3.63 ± 1.16	0.49	6.89	2.00	31	IV		0/0
3. Differentiate NIV indications by disease type	3.89 ± 1.08	3.06 ± 1.01	0.84	10.05	3.26	5	II		12/48
** 4. Explain absolute and relative contraindications to NIV**	**3.82 ± 1.08**	**2.85 ± 1.03**	**0.97**	**10.49**	**3.72**	**4**	**II**	**2nd**	**0/97**
5. Assess level of consciousness, cough ability and secretion management capacity prior to NIV	3.92 ± 1.06	3.32 ± 1.10	0.60	7.43	2.35	27	III		1/0
6. Confirm advance care planning regarding NIV treatment goals and limitations with the patient or caregiver	3.81 ± 1.05	3.02 ± 1.07	0.79	9.06	3.00	14	II		0/9
*B. Equipment setup and application*									
** 7. Explain differences between NIV modes and their respective indications**	**3.82 ± 1.09**	**2.82 ± 0.99**	**1.01**	**10.53**	**3.85**	**2**	**II**	**2nd**	**0/98**
8. Accurately apply initial settings according to physician orders	3.94 ± 1.17	3.19 ± 1.15	0.75	8.27	2.97	15	II		47/12
9. Select the appropriate interface based on patient condition and facial structure	3.82 ± 1.16	3.06 ± 1.17	0.75	7.69	2.88	17	II		0/5
10. Properly fit the interface and secure it at appropriate pressure	3.85 ± 1.17	3.15 ± 1.10	0.70	7.70	2.68	20	II		0/1
11. Understand the clinical significance of circuit leaks and identify abnormal leakage	3.96 ± 1.15	3.28 ± 1.11	0.68	8.11	2.68	21	IV		26/0
12. Systematically verify NIV equipment checklist items	3.84 ± 1.15	3.18 ± 1.10	0.65	7.89	2.51	25	III		0/0
*C. Monitoring*									
13. Regularly monitor vital signs during NIV and recognise abnormal findings	4.20 ± 1.09	3.66 ± 1.19	0.54	7.21	2.28	29	IV		0/0
** 14. Evaluate treatment response within 1–4 h of NIV initiation to determine whether NIV should be continued**	**4.03 ± 1.15**	**3.27 ± 1.19**	**0.75**	**8.94**	**3.04**	**11**	**I**	**2nd**	**90/14**
** 15. Interpret arterial blood gas results to determine the need for NIV setting adjustments**	**4.02 ± 1.11**	**3.25 ± 1.09**	**0.77**	**9.48**	**3.09**	**9**	**I**	**2nd**	**92/22**
** 16. Monitor ventilator waveforms and identify abnormal findings**	**3.95 ± 1.16**	**2.99 ± 1.06**	**0.96**	**10.66**	**3.78**	**3**	**I**	**1st**	**60/98**
17. Assess the adequacy and type of humidification device and determine the need for replacement	3.88 ± 1.15	3.10 ± 1.09	0.78	8.91	3.03	12	II		3/16
18. Observe the amount, viscosity and colour of secretions and intervene to maintain airway clearance	4.08 ± 1.11	3.73 ± 1.14	0.36	5.40	1.47	34	IV		0/0
*D. Complication management*									
19. Identify causes of air leakage and resolve them appropriately	4.06 ± 1.07	3.37 ± 1.08	0.69	8.42	2.80	19	IV		46/2
20. Apply methods to minimise leakage without excessive mask pressure	3.87 ± 1.11	3.12 ± 1.06	0.75	8.66	2.89	16	II		4/6
21. Anticipate the risk of interface-related pressure injury and apply preventive dressings	3.88 ± 1.11	3.52 ± 1.21	0.36	4.70	1.39	35	III		0/0
22. Recognise NIV-related complications such as gastric distension, dry mouth and conjunctival irritation early and intervene	3.90 ± 1.07	3.11 ± 1.08	0.79	6.82	3.08	10	II		34/34
23. Assess causes of patient discomfort or intolerance and apply appropriate interventions	3.88 ± 1.08	3.28 ± 1.06	0.60	8.00	2.32	28	III		0/0
24. Regularly perform oral hygiene for patients on NIV	3.99 ± 1.10	3.61 ± 1.18	0.38	4.73	1.52	33	IV		0/0
*E. Treatment failure and weaning*									
25. Recognise clinical signs indicating NIV treatment failure at an early stage	3.99 ± 1.10	3.34 ± 1.15	0.65	7.47	2.62	22	IV		17/0
26. Promptly report suspected NIV failure to the attending physician and prepare for endotracheal intubation	4.19 ± 1.09	3.61 ± 1.25	0.58	6.83	2.45	26	IV		2/0
** 27. Identify patient–ventilator asynchrony and request appropriate adjustments to NIV settings**	**3.92 ± 1.19**	**2.92 ± 1.14**	**0.99**	**10.33**	**3.89**	**1**	**II**	**2nd**	**20/99**
28. Explain patient characteristics associated with a high risk of NIV failure	3.89 ± 1.15	3.10 ± 1.17	0.80	8.87	3.10	8	II		11/23
29. Identify NIV weaning criteria when the patient’s condition improves and support timely weaning	3.94 ± 1.18	3.15 ± 1.20	0.80	8.28	3.14	7	II		50/30
30. Explain criteria for re-initiation and monitoring methods following NIV weaning	3.92 ± 1.18	3.08 ± 1.21	0.83	9.07	3.25	6	II		19/47
*F. Patient/caregiver education and communication*									
31. Before NIV initiation, explain the treatment purpose, expected discomfort and required cooperation to the patient	3.92 ± 1.15	3.37 ± 1.21	0.55	6.59	2.15	30	III		0/0
32. Provide alternative communication methods for patients undergoing NIV	3.97 ± 1.10	3.55 ± 1.19	0.42	5.44	1.68	32	IV		0/0
33. Educate caregivers on the NIV treatment process, precautions and actions to take when abnormal signs occur	3.94 ± 1.12	3.22 ± 1.14	0.72	8.39	2.83	18	II		35/4
** 34. Accurately and systematically report NIV-related changes in patient status to the care team**	**4.00 ± 1.09**	**3.25 ± 1.19**	**0.75**	**8.53**	**3.01**	**13**	**I**	**2nd**	**90/11**
35. Accurately document NIV-related nursing records	3.94 ± 1.18	3.29 ± 1.21	0.65	7.19	2.55	23	III		11/0

Note: LFF = Locus for Focus model; NIV = non-invasive ventilation; PCL = present competency level (self-rated); RCL = required competency level (perceived importance). All paired *t*-tests: df = 141, *p* < 0.001 (Bonferroni-corrected criterion 0.0014). Quadrants were defined by the grand means of RCL (X_0_ = 3.944) and RCL − PCL (Y_0_ = 0.696), calculated from unrounded item means. Priority (bold): 1st, among the four highest Borich scores and in Quadrant I; 2nd, in one solution only; the classification does not depend on the paired *t*-test. ^a^ Percentage of 5000 bootstrap resamples in Quadrant I/among the top-ranked Borich items (Supplementary Table S2). Full item wording is given in Supplementary File S1.

## Data Availability

The data presented and/or analyzed during the current study are available from the corresponding author upon reasonable request.

## Supplementary Materials

The following supporting information can be downloaded at: https://www.mdpi.com/article/10.3390/healthcare14193326/s1. Supplementary File S1: Educational needs instrument for evidence-based non-invasive ventilation care (English translation and original Korean version); Table S1: Source guideline and revision status of each item of the educational needs instrument for evidence-based NIV care; Table S2: Bootstrap stability of the Borich ranking and the Locus for Focus quadrant allocation for the 35 items (5000 resamples of the 142 participants); Supplementary File S2: STROBE checklist for cross-sectional studies.
