# Supplementary material for: Prioritising Content for Continuing Education in Non-Invasive Ventilation Care: A Needs Assessment Survey of Intensive Care Unit Nurses
Source: Healthcare (Basel). 2026 Oct 6;14(19):3326. doi: 10.3390/healthcare14193326 (PMC13665352; doi:10.3390/healthcare14193326)
Supplement: Supplementary file 1 [file healthcare-14-03326-s001.zip › Supplementary File S1.pdf]

Supplementary File S1.

Educational needs instrument for evidence-based non-invasive ventilation care

(English translation and original Korean version)

The instrument was developed for this study to identify educational priorities for evidence-based non-invasive ventilation (NIV) care. Items were derived from three clinical practice guidelines, consolidated into six domains, and reviewed by an expert panel before administration; the source of each item is given in Supplementary Table S1. The instrument was developed and administered in Korean. The English translation presented with each item was prepared by the research team and checked against the Korean version by a bilingual nursing academic who had not been involved in item development; it is provided for review. Respondents rated every item twice. The required competency level (RCL), a term used in accordance with the Borich needs assessment model, is a rating of the perceived importance of the competency and was rated from 1 (not important at all) to 5 (very important); the present competency level (PCL), a self-rating of present ability to perform the competency, was rated from 1 (not able to perform at all) to 5 (able to perform very well). Item numbers correspond to those used in Table 6 and Figure 1.

Status of the instrument. The instrument was developed for this needs assessment. Its evaluation was limited to expert content validity (I-CVI ≥ .80 for all retained items; S-CVI/Ave = .95) and internal consistency in the study sample (Cronbach's α = .99 for both the RCL and the PCL ratings). Construct validity, factor structure and test–retest reliability have not been examined, and the six domains organise content and are not validated subscales. The instrument is not offered as a validated stand-alone competency scale.

[English Version]

IV. Educational needs for evidence-based non-invasive ventilation care

■ **Instructions**
The following items describe competencies required for NIV care in the intensive care unit. Please respond to both parts of every item.
① Required competency level (RCL; rating of perceived importance): How important is this competency in intensive care unit NIV nursing practice?
② Present competency level (PCL; self-rating of present ability): To what extent are you currently able to perform this competency?

◆ **Response scale**

Scale	1	2	3	4	5
**Required competency level (RCL) : perceived importance**	Not important at all	Not important	Neutral	Important	Very important
**Present competency level (PCL) : self-rated ability**	Not able to perform at all	Mostly not able to perform	Moderately able to perform	Able to perform well	Able to perform very well

A. Indications and pre-application assessment

No	Item	**Required competency level** 1 = not important at all, 5 = very important					**Present competency level** 1 = not able to perform at all, 5 = able to perform very well				
		**1**	**2**	**3**	**4**	**5**	**1**	**2**	**3**	**4**	**5**
1	Identify clinical situations that should prompt consideration of non-invasive ventilation (NIV), such as an increased respiratory rate, use of accessory muscles or deteriorating gas exchange										
2	Interpret arterial blood gas criteria for determining NIV initiation (pH, $PaCO_2$, PaO_2/FiO_2 ratio)										
3	Differentiate NIV indications by disease type (exacerbation of chronic obstructive pulmonary disease, pneumonia in immunocompromised patients, neuromuscular disease, prevention and rescue of post-extubation respiratory failure)										
4	Explain absolute contraindications to NIV (respiratory arrest, inability to protect the airway, haemodynamic instability) and relative contraindications										

No	Item	Required competency level 1 = not important at all, 5 = very important					Present competency level 1 = not able to perform at all, 5 = able to perform very well				
		1	2	3	4	5	1	2	3	4	5
5	Assess level of consciousness, cough ability and secretion management capacity prior to NIV										
6	Confirm advance care planning regarding NIV treatment goals and limitations with the patient or caregiver										

B. Equipment setup and application

No	Item	Required competency level 1 = not important at all, 5 = very important					Present competency level 1 = not able to perform at all, 5 = able to perform very well				
		1	2	3	4	5	1	2	3	4	5
7	Explain differences between NIV modes (continuous positive airway pressure, bilevel positive airway pressure) and their respective indications										
8	Accurately apply initial settings according to physician orders (inspiratory positive airway pressure, expiratory positive airway pressure, respiratory rate, FiO_2)										
9	Select the appropriate interface based on patient condition and facial structure (nasal mask, oronasal mask, full-face mask, helmet)										
10	Properly fit the interface and secure it at appropriate pressure										
11	Understand the clinical significance of circuit leaks and identify abnormal leakage										
12	Systematically verify NIV equipment checklist items (circuit connections, alarm settings, humidifier water level)										

C. Monitoring

No	Item	Required competency level<br>1 = not important at all, 5 = very important					Present competency level<br>1 = not able to perform at all, 5 = able to perform very well				
		1	2	3	4	5	1	2	3	4	5
13	Regularly monitor vital signs during NIV (respiratory rate, pulse, blood pressure, SpO_2) and recognise abnormal findings										
14	Evaluate treatment response within 1–4 hours of NIV initiation (pH, $PaCO_2$, respiratory rate, level of consciousness) to determine whether NIV should be continued										
15	Interpret arterial blood gas results to determine the need for NIV setting adjustments										
16	Monitor ventilator waveforms (pressure, flow and volume graphics) and identify abnormal findings										
17	Assess the adequacy and type of humidification device and determine the need for replacement										
18	Observe the amount, viscosity and colour of secretions and intervene to maintain airway clearance										

D. Complication management

No	Item	Required competency level<br>1 = not important at all, 5 = very important					Present competency level<br>1 = not able to perform at all, 5 = able to perform very well				
		1	2	3	4	5	1	2	3	4	5
19	Identify causes of air leakage (improper mask fitting, excessive airway pressure, mouth opening) and resolve them appropriately										
20	Apply methods to minimise leakage without excessive mask pressure										
21	Anticipate the risk of interface-related pressure injury (skin redness, skin breakdown) and apply preventive dressings										

No	Item	Required competency level<br>1 = not important at all, 5 = very important					Present competency level<br>1 = not able to perform at all, 5 = able to perform very well				
		1	2	3	4	5	1	2	3	4	5
22	Recognise NIV-related complications such as gastric distension, dry mouth and conjunctival irritation early and intervene										
23	Assess causes of patient discomfort or intolerance (mask discomfort, claustrophobia, discomfort from airflow) and apply appropriate interventions										
24	Regularly perform oral hygiene for patients on NIV										

E. Treatment failure and weaning

No	Item	Required competency level<br>1 = not important at all, 5 = very important					Present competency level<br>1 = not able to perform at all, 5 = able to perform very well				
		1	2	3	4	5	1	2	3	4	5
25	Recognise clinical signs indicating NIV treatment failure at an early stage (worsening hypoxaemia, decreased level of consciousness, increased use of accessory muscles, circulatory instability)										
26	Promptly report suspected NIV failure to the attending physician and prepare for endotracheal intubation										
27	Identify patient–ventilator asynchrony and request appropriate adjustments to NIV settings										
28	Explain patient characteristics associated with a high risk of NIV failure (pH < 7.25, severe hypoxaemia, altered consciousness, excessive secretions)										
29	Identify NIV weaning criteria when the patient's condition improves and support timely weaning										
30	Explain criteria for re-initiation and monitoring methods following NIV weaning										

F. Patient/caregiver education and communication

No	Item	Required competency level<br>1 = not important at all, 5 = very important					Present competency level<br>1 = not able to perform at all, 5 = able to perform very well				
		1	2	3	4	5	1	2	3	4	5
31	Before NIV initiation, explain the treatment purpose, expected discomfort and required cooperation to the patient (avoiding mouth breathing, refraining from removing the mask)										
32	Provide alternative communication methods for patients undergoing NIV (writing, hand signals)										
33	Educate caregivers on the NIV treatment process, precautions and actions to take when abnormal signs occur										
34	Accurately and systematically report NIV-related changes in patient status (treatment response, complications, signs of failure) to the care team										
35	Accurately document NIV-related nursing records (settings, monitoring results, complications, education provided)										

[Korean Version]

IV. 근거기반 비침습적 인공호흡 간호 교육요구도

■ 작성 안내

다음 문항은 중환자실 NIV 간호에 필요한 역량을 제시한 것입니다. 각 문항에 대해 **두 가지 항목**에 모두 응답해 주십시오.

① **중요도(RCL):** 해당 역량이 중환자실 NIV 간호 실무에서 얼마나 중요한가?

② **현재 수행 수준(PCL):** 현재 본인이 해당 역량을 어느 정도 수행할 수 있는가?

◆ 응답 척도

구분	1 점	2 점	3 점	4 점	5 점
중요도 (RCL)	전혀 중요하지 않다	중요하지 않다	보통이다	중요하다	매우 중요하다
현재 수행 수준 (PCL)	전혀 수행하지 못한다	대부분 수행하지 못한다	보통이다	잘 수행한다	매우 잘 수행한다

영역 1. 적응증 및 적용 전 사정

번호	문 항	중요도 (RCL) 전혀 중요하지 않다(1) ~매우 중요하다(5)					현재 수행 수준 (PCL) 전혀 수행하지 못한다(1) ~매우 잘 수행한다(5)				
		1	2	3	4	5	1	2	3	4	5
1	비침습적 인공호흡(NIV)의 적용을 고려해야 하는 임상 상황(호흡수 증가, 보조근 사용, 가스교환 악화 등)을 파악할 수 있다										
2	NIV 적용 여부를 판단하기 위한 동맥혈가스 기준(pH, $PaCO_2$, PaO_2/FiO_2 ratio)을 해석할 수 있다										
3	질환별(COPD 악화, 면역저하 환자의 폐렴, 신경근육질환, 발관 후 호흡부전 예방 및 구제 등) NIV 적응증을 구분할 수 있다										

번호	문 항	중요도 (RCL)<br>전혀 중요하지 않다(1)<br>~매우 중요하다(5)					현재 수행 수준 (PCL)<br>전혀 수행하지 못한다(1)<br>~매우 잘 수행한다(5)				
		1	2	3	4	5	1	2	3	4	5
4	NIV 의 절대적 금기(호흡 정지, 기도 보호 불능, 혈역학적 불안정 등)와 상대적 금기를 설명할 수 있다										
5	NIV 적용 전 환자의 의식 수준, 기침 능력 및 분비물 관리 능력을 사정할 수 있다										
6	환자 또는 보호자와 NIV 치료 목표와 한계에 대한 사전 계획을 확인할 수 있다										

영역 2. 장비 준비 및 적용

번호	문 항	중요도 (RCL)					현재 수행 수준 (PCL)				
		1	2	3	4	5	1	2	3	4	5
7	NIV 모드(CPAP, bilevel/BiPAP)의 차이와 각 모드의 적응증을 설명할 수 있다										
8	처방에 따라 IPAP, EPAP, 호흡수, FiO_2 등 초기 설정값을 정확히 적용할 수 있다										
9	환자 상태와 얼굴 구조에 따라 적절한 인터페이스(비강마스크, 구비강마스크, 전안면마스크, 헬멧 등)를 선택할 수 있다										
10	인터페이스를 올바르게 장착하고 적절한 압력으로 고정할 수 있다										
11	회로 누출의 임상적 의미를 이해하고 비정상 누출을 인지할 수 있다										
12	NIV 장비 점검 항목(회로 연결 상태, 알람 설정, 가습기 수위 등)을 체계적으로 확인할 수 있다										

영역 3. 모니터링

번호	문 항	중요도 (RCL)					현재 수행 수준 (PCL)				
		1	2	3	4	5	1	2	3	4	5
13	NIV 적용 중 활력징후(호흡수, 맥박, 혈압, SpO_2)를 정기적으로 모니터링하고 이상 소견을 인지할 수 있다										
14	NIV 시작 후 1~4 시간 내 치료반응(pH, $PaCO_2$, 호흡수, 의식수준)을 평가하여 치료 지속 여부를 판단할 수 있다										
15	동맥혈가스 결과를 해석하여 NIV 설정 조정의 필요성을 판단할 수 있다										
16	환기기 파형(압력, 유량, 용량 그래픽)을 모니터링하고 비정상 소견을 파악할 수 있다										
17	가습 장치의 적절성과 유형을 사정하고 필요 시 교체 여부를 판단할 수 있다										
18	분비물의 양, 점도, 색깔을 관찰하고 기도 청결 유지를 위한 적절한 중재를 시행할 수 있다										

영역 4. 합병증 관리

번호	문 항	중요도 (RCL)					현재 수행 수준 (PCL)				
		1	2	3	4	5	1	2	3	4	5
19	공기누출의 원인(마스크 부적절한 장착, 과도한 기도 압력, 구강 열림 등)을 파악하고 적절한 방법으로 해결할 수 있다										
20	마스크 과도 압박 없이 누출을 최소화하는 방법을 적용할 수 있다										
21	NIV 인터페이스로 인한 압력손상(피부 발적, 피부 손상 등) 위험을 예측하고 예방적 드레싱을 적용할 수 있다										

번호	문 항	중요도 (RCL)					현재 수행 수준 (PCL)				
		1	2	3	4	5	1	2	3	4	5
22	위 팽만, 구강 건조, 결막 자극 등 NIV 관련 합병증을 조기에 인식하고 중재할 수 있다										
23	환자의 불편감이나 불내성(마스크 불편감, 폐소공포증, 공기 흐름 불편 등) 원인을 사정하고 적절한 중재를 적용할 수 있다										
24	NIV 적용 중 구강 위생을 정기적으로 수행할 수 있다										

영역 5. 치료 실패 및 이탈

번호	문 항	중요도 (RCL)					현재 수행 수준 (PCL)				
		1	2	3	4	5	1	2	3	4	5
25	NIV 치료 실패를 시사하는 임상 징후(저산소혈증 악화, 의식 저하, 호흡 보조근 사용 증가, 순환 불안정 등)를 조기에 인식할 수 있다										
26	NIV 치료 실패가 의심될 때 즉각적으로 담당 의사에게 보고하고 기관내 삽관 시행 준비를 할 수 있다										
27	환자-기계 비동조(asynchrony) 상황을 파악하고 이에 따른 설정 조정을 요청할 수 있다										
28	NIV 치료 실패 가능성이 높은 환자 특성($pH < 7.25$, 중증 저산소혈증, 의식 변화, 과다 분비물 등)을 설명할 수 있다										
29	환자 상태 호전 시 NIV 이탈(weaning) 기준을 파악하고 적절한 시기에 이탈을 지원할 수 있다										
30	NIV 이탈 후 재적용 기준과 모니터링 방법을 설명할 수 있다										

영역 6. 환자·보호자 교육 및 의사소통

번호	문 항	중요도 (RCL)					현재 수행 수준 (PCL)				
		1	2	3	4	5	1	2	3	4	5
31	NIV 적용 전 치료 목적, 예상되는 불편감, 협조 방법(구강 호흡 억제, 마스크 제거 자제 등)을 환자에게 설명할 수 있다										
32	환자가 NIV 치료 중 의사소통할 수 있는 대안적 방법(글쓰기, 신호 등)을 제공할 수 있다										
33	보호자에게 NIV 치료 과정, 주의사항 및 이상 징후 발생 시 대처 방법을 교육할 수 있다										
34	NIV 관련 환자 상태 변화(치료반응, 합병증, 실패 징후)를 의료팀에 정확하고 체계적으로 보고할 수 있다										
35	NIV 관련 간호 기록(설정값, 모니터링 결과, 합병증, 교육 내용 등)을 정확하게 작성할 수 있다										
