# Supplementary material for: Prioritising Content for Continuing Education in Non-Invasive Ventilation Care: A Needs Assessment Survey of Intensive Care Unit Nurses
Source: Healthcare (Basel). 2026 Oct 6;14(19):3326. doi: 10.3390/healthcare14193326 (PMC13665352; doi:10.3390/healthcare14193326)
Supplement: Supplementary file 1 [file healthcare-14-03326-s001.zip › Supplementary File S2. STROBE checklist Ethical approval document.pdf]

STROBE Statement—Checklist of items that should be included in reports of ***cross-sectional studies***

Manuscript ID: healthcare-4562366.R1

Title: Prioritising content for continuing education in non-invasive ventilation care: A needs assessment survey of intensive care unit nurses

	Item No	Recommendation	Page No
**Title and abstract**	1	(*a*) Indicate the study's design with a commonly used term in the title or the abstract	Title: p. 1, lines 2–4; Abstract, Methods: p. 1, line 18
		(*b*) Provide in the abstract an informative and balanced summary of what was done and what was found	Abstract: p. 1, lines 12–37
**Introduction**			
Background/rationale	2	Explain the scientific background and rationale for the investigation being reported	Section 1, paragraphs 1–5: p. 1–3, lines 38–94
Objectives	3	State specific objectives, including any prespecified hypotheses	Section 1, paragraph 6: p. 3, lines 95–103 No hypotheses were prespecified.
**Methods**			
Study design	4	Present key elements of study design early in the paper	Abstract: p. 1, line 18; Section 2.1: p. 3, lines 105–108
Setting	5	Describe the setting, locations, and relevant dates, including periods of recruitment, exposure, follow-up, and data collection	Section 2.2, paragraph 1: p. 3–4, lines 109-133; Section 2.4: p. 6, lines 234-242
Participants	6	(*a*) Give the eligibility criteria, and the sources and methods of selection of participants	Section 2.2, paragraph 1: p. 3–4, lines 117–123; Section 3.1: p. 8, lines 332–334
Variables	7	Clearly define all outcomes, exposures, predictors, potential confounders, and effect modifiers. Give diagnostic criteria, if applicable	Sections 2.3.1–2.3.5: p. 4–6, lines 141–233
Data sources/ measurement	8*	For each variable of interest, give sources of data and details of methods of assessment (measurement). Describe comparability of assessment methods if there is more than one group	Sections 2.3.1–2.3.4: p. 4–6, lines 141–228; Section 3.2, paragraph 1: p. 9, lines 350–368. All data were self-reported on a single structured questionnaire administered identically to every participant.
Bias	9	Describe any efforts to address potential sources of bias	Section 2.2: p. 3, lines 120–123; Section 2.4: p. 6, lines 239–242; Section 2.6: p. 7-8,

			lines 323–329; Section 4.2, paragraph 1: p. 18-19, lines 667–710
Study size	10	Explain how the study size was arrived at	Section 2.2, paragraph 2: p. 3, lines 124–133
Quantitative variables	11	Explain how quantitative variables were handled in the analyses. If applicable, describe which groupings were chosen and why	Section 2.5, paragraph 3: p. 7, lines 274–287; Tables 1 and 3
Statistical methods	12	(*a*) Describe all statistical methods, including those used to control for confounding	Section 2.5: p. 6–8, lines 244–316; multivariable adjustment: Table 4
		(*b*) Describe any methods used to examine subgroups and interactions	Section 2.5, paragraph 3: p. 7, lines 274–287
		(*c*) Explain how missing data were addressed	Section 2.5, paragraph 1: p. 6, lines 245–250; Section 3.2, paragraph 2: p. 9, lines 359–363
		(*d*) If applicable, describe analytical methods taking account of sampling strategy	Not applicable. Convenience sampling from a single centre; no design-based weighting or clustering adjustment was required. Nesting of nurses within units was examined by ICC(1): Section 3.5, p. 11, lines 434–435.
		(*e*) Describe any sensitivity analyses	Section 2.5, paragraph 6: p. 7, lines 311–316; Section 3.7, paragraph 3: p. 13, lines 476–488; Section 3.2, paragraphs 2–3: p. 9, lines 359–368
**Results**			
Participants	13*	(a) Report numbers of individuals at each stage of study—eg numbers potentially eligible, examined for eligibility, confirmed eligible, included in the study, completing follow-up, and analysed	Section 2.2: p. 3, line 119-120; Section 3.1: p. 8, lines 332–334
		(b) Give reasons for non-participation at each stage	Section 3.1: p. 8, lines 332–334
		(c) Consider use of a flow diagram	Not applicable. A flow diagram was not included; all numbers are reported in the text.
Descriptive data	14*	(a) Give characteristics of study participants (eg demographic, clinical, social) and information on exposures and potential confounders	Section 3.1: p. 8–9, lines 334–349; Table 1

		(b) Indicate number of participants with missing data for each variable of interest	Section 3.2, paragraph 2: p. 9, lines 359–363
Outcome data	15*	Report numbers of outcome events or summary measures	Sections 3.3–3.7: p. 9–15, lines 369–505; Tables 2–6; Figure 1
Main results	16	(*a*) Give unadjusted estimates and, if applicable, confounder-adjusted estimates and their precision (eg, 95% confidence interval). Make clear which confounders were adjusted for and why they were included	Section 3.4 and Table 3 (unadjusted estimates with 95% CIs): p. 10-11, lines 387–423; Section 3.5 and Table 4 (adjusted estimates): p. 11, lines 424–451; covariates and rationale: Section 2.5, paragraph 4, p. 7, lines 288–310
		(*b*) Report category boundaries when continuous variables were categorized	Section 2.5, paragraph 3: p. 6, lines 260–264; Tables 1 and 3
		(*c*) If relevant, consider translating estimates of relative risk into absolute risk for a meaningful time period	Not applicable.
Other analyses	17	Report other analyses done—eg analyses of subgroups and interactions, and sensitivity analyses	Section 3.4, paragraph 3 (Holm adjustment): p. 10, lines 410–414; Section 3.5 (multivariable models, ICC): p. 11, lines 425–435; Section 3.7, paragraph 3 (bootstrap): p. 13, lines 476–488; Table 6; Figure 1; Supplementary Table S2
**Discussion**			
Key results	18	Summarise key results with reference to study objectives	Section 4, paragraph 1: p. 15, lines 507–514; Section 5: p. 19-20, lines 712–732
Limitations	19	Discuss limitations of the study, taking into account sources of potential bias or imprecision. Discuss both direction and magnitude of any potential bias	Section 4.2: p. 18-19, lines 668–710
Interpretation	20	Give a cautious overall interpretation of results considering objectives, limitations, multiplicity of analyses, results from similar studies, and other relevant evidence	Sections 4 and 4.1: p. 15–19, lines 514–710
Generalisability	21	Discuss the generalisability (external validity) of the study results	Section 4.2, paragraph 3: p. 19,

			lines 708–710
**Other information**			
Funding	22	Give the source of funding and the role of the funders for the present study and, if applicable, for the original study on which the present article is based	Funding statement: p. 20, line 745-747

Note: An Explanation and Elaboration article discusses each checklist item and gives methodological background and published examples of transparent reporting. The STROBE checklist is best used in conjunction with this article (freely available on the Web sites of PLoS Medicine at http://www.plosmedicine.org/, Annals of Internal Medicine at http://www.annals.org/, and Epidemiology at http://www.epidem.com/). Information on the STROBE Initiative is available at www.strobe-statement.org.

Ethical statement

IRB approval (Korean version)

DocNo : CHOSUN202604024-HE002

통지서

QR코드를 읽어 문서 진위여부를 확인할 수 있습니다.

※ 본 과제의 문서보존기간은 3년입니다.

수신	지원(의뢰)기관	내부과제			
	연구책임자	정승은 / 조선대학교병원 / 특수부서 간호팀/내과계 중환자실			
File No.	CHOSUN IRB 2026-04-024	신청유형	신규신청서	통지일자	2026.05.06
과제명	국문	중환자실 간호사의 비침습적 인공호흡 간호역량, 실행 문화 및 근거기반 비침습적 인공호흡 간호 교육 요구도 우선순위			
	영문	Competence in Noninvasive Ventilation Nursing, Implementation Climate, and Prioritized Educational Needs for Evidence-Based Noninvasive Ventilation Nursing Among Intensive Care Unit Nurses			
Study Nick Name					

연구구분	임상시험 외 연구		
대상자 수	전체	130명	
	국내	130명	
	본원	130명	
연구승인기간	2026-05-06 ~ 2027-05-05		
지원(의뢰) 기관	내부과제		
제출서류 목록	1. 계획서 요약<br>2. 피험자 동의서 (Informed Consent Form)<br>3. 피험자 모집관련 공고(공고문, 매체정보 등)<br>4. 연구계획서<br>5. 연구자 최근 이력서 및 IRB교육이력<br>6. 이해상충 서약서<br>7. 연구대상자 설명문<br>8. 생명윤리준수서약서		
심사일	2026.05.06	심사종류	신속심사
중간보고 시기	2027년 03월 05일부터	비고	
심사결과	● 승인 ○ 시정승인		
내용	A. 기본 평가<br>1) 신청유형: 신규<br>2) 연구책임자(간호부 정승은 선생님)는 본 연구를 수행하기에 적절한 자격과 경험을 갖추었다고 판단합니다.<br><br>B. 상세 평가<br>3) 본 연구는 " 중환자실 간호사의 비침습적 인공호흡 간호역량, 실행 문화 및 근거기반 비침습적 인공호흡 간호 교육 요구도 우선순위" 설문조사 연구입니다.<br>4) 본 연구의 위험도는 최소위험(minimal risk)에 해당됩니다.<br>5) 본 연구는 피험자가 겪을 수 있는 위험(risks)에 대해 충분히 파악되어 있으며, 이를 최소화하기 위해 적절한 조치가 되어 있습니다.<br>6) 본 연구는 피험자가 겪을 수 있는 위험(risk)이 잠재적 이익(benefit)보다 크지 않습니다.<br>7) 연구대상자의 안전 및 개인정보보호 방안에 대한 기술은 적절합니다.<br>8) 연구대상자 모집과 선정과정은 적절합니다.<br>9) 연구대상자 설명문 및 동의 절차는 적절합니다.<br>10) 취약한 연구대상자의 보호대책은 적절합니다.<br>11) 본 연구의 위험수준을 고려하였을 때, 지속심사주기는 12개월 입니다.<br><br>*주의사항 : 연구자(시험자)를 포함한 모든 참여인력은 당해년도에 1회 이상 필수교육을 이수하여야 합니다.		

1 개정본 2024.07.01

DocNo : CHOSUN202604024-HE002

별첨	

조선대학교병원 기관생명윤리위원회 위원장

(English version)

Ethical Statement

Study title: Prioritising content for continuing education in non-invasive ventilation care: A needs assessment survey of intensive care unit nurses

The study was reviewed and approved by the Institutional Review Board of Chosun University Hospital, Republic of Korea.

- Approval number: CHOSUN IRB 2026-04-024
- Type of Review: Full Board Review / Expedited Review
- Review Result: Approved
- Approval Date: May 6, 2026
- Study Period: May 6, 2026 to May 5, 2027
- Institutional authority: Chairperson, Institutional Review Board of Chosun University Hospital

The study involved anonymous voluntary survey participation. Written informed consent was obtained from all participants prior to data collection.
