# Supplementary material for: Prioritising Content for Continuing Education in Non-Invasive Ventilation Care: A Needs Assessment Survey of Intensive Care Unit Nurses
Source: Healthcare (Basel). 2026 Oct 6;14(19):3326. doi: 10.3390/healthcare14193326 (PMC13665352; doi:10.3390/healthcare14193326)
Supplement: Supplementary file 1 [file healthcare-14-03326-s001.zip › Supplementary Tables S1 and S2.pdf]

Supplementary Materials (Supplementary Tables S1, S2)

Table S1. Source guideline and revision status of each item of the educational needs instrument for evidence-based NIV care

Item (final wording)	Source guideline	Recommendation or section informing the item	Wording revised after expert review	Preliminary wording before revision (English translation)
**A. Indications and pre-application assessment**				
1. Identify clinical situations that should prompt consideration of non-invasive ventilation (NIV), such as an increased respiratory rate, use of accessory muscles or deteriorating gas exchange	ERS/ATS guideline on NIV for acute respiratory failure [3]	Indications; clinical criteria for considering NIV	No	–
2. Interpret arterial blood gas criteria for determining NIV initiation (pH, $PaCO_2$, PaO_2/FiO_2 ratio)	ERS/ATS guideline on NIV for acute respiratory failure [3]	Indications; arterial blood gas criteria for acute hypercapnic and hypoxaemic respiratory failure	Yes	Interpret arterial blood gas criteria for determining NIV initiation (pH, $PaCO_2$)
3. Differentiate NIV indications by disease type (exacerbation of chronic obstructive pulmonary disease, pneumonia in immunocompromised patients, neuromuscular disease, prevention and rescue of post-extubation respiratory failure)	ERS/ATS guideline on NIV for acute respiratory failure [3]	Disease-specific recommendations (COPD exacerbation, immunocompromised patients, post-extubation, neuromuscular disease)	Yes	Differentiate NIV indications by disease (COPD exacerbation, cardiogenic pulmonary oedema, pneumonia in immunocompromised patients)
4. Explain absolute contraindications to NIV (respiratory arrest, inability to protect the airway, haemodynamic instability) and relative contraindications	ERS/ATS guideline on NIV for acute respiratory failure [3]; NSW ACI clinical practice guide for NIV in acute respiratory failure [21]	Contraindications to NIV	No	–
5. Assess level of consciousness, cough ability and secretion management capacity prior to NIV	NSW ACI clinical practice guide for NIV in acute respiratory failure [21]	Pre-application patient assessment (consciousness, airway protection, secretions)	No	–
6. Confirm advance care planning regarding NIV treatment goals and limitations with the patient or caregiver	ERS/ATS guideline on NIV for acute respiratory failure [3]; NSW ACI clinical practice guide for NIV in acute respiratory failure [21]	Palliative NIV and treatment goals; ceiling-of-treatment discussion	No	–
**B. Equipment setup and application**				
7. Explain differences between NIV modes (continuous positive airway pressure, bilevel positive airway pressure) and their respective indications	NSW ACI clinical practice guide for NIV in acute respiratory failure [21]	Modes of NIV (CPAP, bilevel) and their indications	No	–
8. Accurately apply initial settings according to physician orders (inspiratory positive airway pressure, expiratory positive airway pressure, respiratory rate, FiO_2)	NSW ACI clinical practice guide for NIV in acute respiratory failure [21]	Initial settings (IPAP, EPAP, respiratory rate, FiO_2)	No	–

9. Select the appropriate interface based on patient condition and facial structure (nasal mask, oronasal mask, full-face mask, helmet)	NSW ACI clinical practice guide for NIV in acute respiratory failure [21]; AARC guideline on patient–ventilator assessment [20]	Interface selection; interface inspection	No	–
10. Properly fit the interface and secure it at appropriate pressure	NSW ACI clinical practice guide for NIV in acute respiratory failure [21]; AARC guideline on patient–ventilator assessment [20]	Interface fitting and strap tension; interface inspection	No	–
11. Understand the clinical significance of circuit leaks and identify abnormal leakage	AARC guideline on patient–ventilator assessment [20]	Assessment of circuit and interface leak	Yes	Determine whether a leak port is required according to the circuit type (single or double circuit) and assemble the appropriate circuit
12. Systematically verify NIV equipment checklist items (circuit connections, alarm settings, humidifier water level)	AARC guideline on patient–ventilator assessment [20]	Assessment of the ventilator circuit, alarms and humidifier	No	–
**C. Monitoring**				
13. Regularly monitor vital signs during NIV (respiratory rate, pulse, blood pressure, SpO_2) and recognise abnormal findings	NSW ACI clinical practice guide for NIV in acute respiratory failure [21]	Monitoring during NIV (vital signs, SpO_2)	No	–
14. Evaluate treatment response within 1–4 hours of NIV initiation (pH, $PaCO_2$, respiratory rate, level of consciousness) to determine whether NIV should be continued	ERS/ATS guideline on NIV for acute respiratory failure [3]; NSW ACI clinical practice guide for NIV in acute respiratory failure [21]	Reassessment of response within the first hours of NIV	Yes	Evaluate treatment response within 1–4 hours of NIV initiation (pH, $PaCO_2$, change in respiratory rate) to determine whether NIV should be continued
15. Interpret arterial blood gas results to determine the need for NIV setting adjustments	NSW ACI clinical practice guide for NIV in acute respiratory failure [21]	Arterial blood gas monitoring and adjustment of settings	No	–
16. Monitor ventilator waveforms (pressure, flow and volume graphics) and identify abnormal findings	AARC guideline on patient–ventilator assessment [20]	Assessment of ventilator graphics (pressure, flow, volume)	No	–
17. Assess the adequacy and type of humidification device and determine the need for replacement	AARC guideline on patient–ventilator assessment [20]	Assessment of the adequacy of humidification	Yes	Assess the adequacy and type of humidification device (heated humidifier or heat–moisture exchanger) and determine the need for replacement
18. Observe the amount, viscosity and colour of secretions and intervene to maintain airway clearance	NSW ACI clinical practice guide for NIV in acute respiratory failure [21]	Secretion management and airway clearance	No	–
**D. Complication management**				
19. Identify causes of air leakage (improper mask fitting, excessive airway pressure, mouth opening) and resolve them appropriately	AARC guideline on patient–ventilator assessment [20]; NSW ACI clinical practice guide for NIV in acute respiratory failure [21]	Leak assessment; troubleshooting of leaks	No	–

20. Apply methods to minimise leakage without excessive mask pressure	NSW ACI clinical practice guide for NIV in acute respiratory failure [21]	Minimising leak without excessive mask pressure	Yes	Apply methods to minimise leakage without excessive mask pressure (adjusting mask straps, adjusting rise time)
21. Anticipate the risk of interface-related pressure injury (skin redness, skin breakdown) and apply preventive dressings	NSW ACI clinical practice guide for NIV in acute respiratory failure [21]	Prevention of interface-related pressure injury	No	–
22. Recognise NIV-related complications such as gastric distension, dry mouth and conjunctival irritation early and intervene	NSW ACI clinical practice guide for NIV in acute respiratory failure [21]	Recognition and management of complications (gastric distension, dryness, eye irritation)	No	–
23. Assess causes of patient discomfort or intolerance (mask discomfort, claustrophobia, discomfort from airflow) and apply appropriate interventions	NSW ACI clinical practice guide for NIV in acute respiratory failure [21]	Management of intolerance and discomfort	No	–
24. Regularly perform oral hygiene for patients on NIV	NSW ACI clinical practice guide for NIV in acute respiratory failure [21]	Oral care during NIV	Yes	Regularly (every 2 hours) perform oral hygiene for patients on NIV
**E. Treatment failure and weaning**				
25. Recognise clinical signs indicating NIV treatment failure at an early stage (worsening hypoxaemia, decreased level of consciousness, increased use of accessory muscles, circulatory instability)	ERS/ATS guideline on NIV for acute respiratory failure [3]; NSW ACI clinical practice guide for NIV in acute respiratory failure [21]	Recognition of NIV failure	No	–
26. Promptly report suspected NIV failure to the attending physician and prepare for endotracheal intubation	ERS/ATS guideline on NIV for acute respiratory failure [3]; NSW ACI clinical practice guide for NIV in acute respiratory failure [21]	Escalation and preparation for intubation when NIV fails	Yes	Promptly report suspected NIV failure to the attending physician and prepare for endotracheal intubation
27. Identify patient–ventilator asynchrony and request appropriate adjustments to NIV settings	NSW ACI clinical practice guide for NIV in acute respiratory failure [21]; AARC guideline on patient–ventilator assessment [20]	Patient–ventilator asynchrony	Yes	Identify patient–ventilator asynchrony (double triggering, auto-triggering, ineffective triggering) and inform the team of the need for setting adjustment according to its cause
28. Explain patient characteristics associated with a high risk of NIV failure (pH < 7.25, severe hypoxaemia, altered consciousness, excessive secretions)	ERS/ATS guideline on NIV for acute respiratory failure [3]	Predictors of NIV failure	No	–
29. Identify NIV weaning criteria when the patient's condition improves and support timely weaning	NSW ACI clinical practice guide for NIV in acute respiratory failure [21]	Weaning from NIV	No	–
30. Explain criteria for re-initiation and monitoring methods following NIV weaning	NSW ACI clinical practice guide for NIV in acute respiratory failure [21]	Monitoring and re-initiation after weaning	No	–
**F. Patient/caregiver education and communication**				
31. Before NIV initiation, explain the treatment purpose, expected discomfort and required cooperation to the	NSW ACI clinical practice guide for NIV in acute respiratory failure [21]	Patient explanation before NIV	No	–

patient (avoiding mouth breathing, refraining from removing the mask)				
32. Provide alternative communication methods for patients undergoing NIV (writing, hand signals)	NSW ACI clinical practice guide for NIV in acute respiratory failure [21]	Communication with the patient during NIV	No	–
33. Educate caregivers on the NIV treatment process, precautions and actions to take when abnormal signs occur	NSW ACI clinical practice guide for NIV in acute respiratory failure [21]	Caregiver information	No	–
34. Accurately and systematically report NIV-related changes in patient status (treatment response, complications, signs of failure) to the care team	NSW ACI clinical practice guide for NIV in acute respiratory failure [21]	Escalation and communication of deterioration to the treating team	No	–
35. Accurately document NIV-related nursing records (settings, monitoring results, complications, education provided)	NSW ACI clinical practice guide for NIV in acute respiratory failure [21]; AARC guideline on patient–ventilator assessment [20]	Documentation of settings, monitoring and care	No	–
Deleted preliminary item (Domain F): Participate, as a member of the multidisciplinary team, in difficult conversations about the limits and goals of NIV treatment (e.g., refusal of intubation, palliative NIV)	ERS/ATS guideline on NIV for acute respiratory failure [3]; NSW ACI clinical practice guide for NIV in acute respiratory failure [21]	Palliative NIV; goals-of-care discussion	Deleted (I-CVI < .78)	–

Note: AARC = American Association for Respiratory Care; ACI = Agency for Clinical Innovation; ERS/ATS = European Respiratory Society/American Thoracic Society; I-CVI = item-level content validity index. Reference numbers refer to the reference list of the main text. Items were retained when I-CVI ≥ .78 [22]; the I-CVI of the retained items is summarised in Section 3.2. The wording of nine items was revised in response to panel comments, mainly by removing examples that the panel considered too specific or setting-dependent (items 17, 20, 24, 27), by aligning the clinical examples with the ERS/ATS recommendations (items 2, 3, 14) and by replacing a circuit-configuration task with the recognition of abnormal leakage (item 11); the change to item 26 was a minor clarification. Item numbers are those of the final instrument; the deleted item was preliminary item 35, and preliminary item 36 became final item 35. The six domains organise content and are not psychometric subscales.

Table S2. Bootstrap stability of the Borich ranking and the Locus for Focus quadrant allocation for the 35 items (5000 resamples of the 142 participants)

Domain/ item	Borich rank (full sample)	LFF quadrant (full sample)	In Quadrant I (%)	Among top-k Borich items (%)	In both solutions (%)	In either solution (%)	Borich rank: median (2.5th–97.5th percentile)
**A. Indications and pre-application assessment**							
1. Identify clinical situations that should prompt consideration of NIV	24	III	5.2	0.1	0.1	5.3	24 (14–30)
2. Interpret arterial blood gas criteria for determining NIV initiation	31	IV	0.0	0.0	0.0	0.0	31 (26–34)
3. Differentiate NIV indications by disease type	5	II	11.9	47.5	9.7	49.8	7 (4–16)
**4. Explain absolute and relative contraindications to NIV**	**4**	**II**	**0.0**	**96.6**	**0.0**	**96.6**	**3 (1–7)**
5. Assess level of consciousness, cough ability and secretion management capacity prior to NIV	27	III	1.3	0.0	0.0	1.3	27 (18–31)
6. Confirm advance care planning regarding NIV treatment goals and limitations with the patient or caregiver	14	II	0.0	8.6	0.0	8.6	13 (6–21)
**B. Equipment setup and application**							
**7. Explain differences between NIV modes and their respective indications**	**2**	**II**	**0.0**	**98.3**	**0.0**	**98.3**	**2 (1–6)**
8. Accurately apply initial settings according to physician orders	15	II	46.9	12.0	9.6	49.3	13 (5–23)
9. Select the appropriate interface based on patient condition and facial structure	17	II	0.1	4.9	0.1	4.9	16 (6–25)
10. Properly fit the interface and secure it at appropriate pressure	20	II	0.2	0.7	0.0	0.9	21 (10–28)
11. Understand the clinical significance of circuit leaks and identify abnormal leakage	21	IV	25.5	0.2	0.2	25.5	21 (12–27)
12. Systematically verify NIV equipment checklist items	25	III	0.0	0.0	0.0	0.0	24 (16–29)
**C. Monitoring**							
13. Regularly monitor vital signs during NIV and recognise abnormal findings	29	IV	0.2	0.0	0.0	0.2	28 (20–31)
**14. Evaluate treatment response within 1–4 hours of NIV initiation to determine whether NIV should be continued**	**11**	**I**	**90.1**	**13.6**	**13.6**	**90.1**	**12 (6–21)**
**15. Interpret arterial blood gas results to determine the need for NIV setting adjustments**	**9**	**I**	**91.9**	**22.0**	**22.0**	**91.9**	**11 (5–21)**
**16. Monitor ventilator waveforms and identify abnormal findings**	**3**	**I**	**59.5**	**97.9**	**59.4**	**98.0**	**3 (1–5)**
17. Assess the adequacy and type of humidification device and determine the need for replacement	12	II	3.4	16.1	2.1	17.3	12 (5–22)
18. Observe the amount, viscosity and colour of secretions and intervene to maintain airway clearance	34	IV	0.0	0.0	0.0	0.0	34 (32–35)

**D. Complication management**							
19. Identify causes of air leakage and resolve them appropriately	19	IV	45.5	2.3	2.3	45.5	18 (9–26)
20. Apply methods to minimise leakage without excessive mask pressure	16	II	3.9	5.6	1.3	8.1	16 (6–25)
21. Anticipate the risk of interface-related pressure injury and apply preventive dressings	35	III	0.0	0.0	0.0	0.0	34 (32–35)
22. Recognise NIV-related complications such as gastric distension, dry mouth and conjunctival irritation early and intervene	10	II	33.9	34.0	27.9	40.0	11 (1–29)
23. Assess causes of patient discomfort or intolerance and apply appropriate interventions	28	III	0.3	0.0	0.0	0.3	27 (20–31)
24. Regularly perform oral hygiene for patients on NIV	33	IV	0.0	0.0	0.0	0.0	33 (31–35)
**E. Treatment failure and weaning**							
25. Recognise clinical signs indicating NIV treatment failure at an early stage	22	IV	17.2	0.3	0.3	17.2	22 (13–28)
26. Promptly report suspected NIV failure to the attending physician and prepare for endotracheal intubation	26	IV	2.1	0.1	0.1	2.1	25 (15–31)
**27. Identify patient–ventilator asynchrony and request appropriate adjustments to NIV settings**	**1**	**II**	**19.9**	**99.0**	**19.9**	**99.0**	**2 (1–5)**
28. Explain patient characteristics associated with a high risk of NIV failure	8	II	10.6	23.1	6.2	27.5	10 (5–20)
29. Identify NIV weaning criteria when the patient's condition improves and support timely weaning	7	II	50.0	29.8	24.2	55.6	9 (4–20)
30. Explain criteria for re-initiation and monitoring methods following NIV weaning	6	II	18.6	47.3	15.6	50.4	7 (4–14)
**F. Patient/caregiver education and communication**							
31. Before NIV initiation, explain the treatment purpose, expected discomfort and required cooperat…	30	III	0.1	0.0	0.0	0.1	29 (23–31)
32. Provide alternative communication methods for patients undergoing NIV	32	IV	0.0	0.0	0.0	0.0	32 (30–35)
33. Educate caregivers on the NIV treatment process, precautions and actions to take when abnormal signs occur	18	II	35.0	3.7	3.4	35.3	17 (8–26)
**34. Accurately and systematically report NIV-related changes in patient status to the care team**	**13**	**I**	**90.5**	**11.3**	**11.3**	**90.5**	**12 (6–20)**
35. Accurately document NIV-related nursing records	23	III	11.3	0.2	0.2	11.3	23 (14–29)

Note: LFF = Locus for Focus. Item means, discrepancies and Borich scores are given in Table 6 of the main text. In each of 5000 resamples drawn with replacement from the 142 participants, the grand means of RCL and RCL − PCL, the quadrant allocation, the Borich ranking and k, the number of items in Quadrant I, were recalculated. "Top-k" denotes the k highest-ranked Borich items in that resample (k ranged from 1 to 13; median 7). "Both solutions" denotes membership of Quadrant I and of the top-k Borich items in the same resample, the criterion for the first priority; "either solution" denotes membership of at least one. The seven priority candidates of the main analysis (items 4, 7, 14, 15, 16, 27 and 34) are in bold.
